# Mosquito-bite infection of humanized mice with chikungunya virus produces systemic disease with long-term effects

**DOI:** 10.1371/journal.pntd.0009427

**Published:** 2021-06-09

**Authors:** Brianne M. Hibl, Natalie J. M. Dailey Garnes, Alexander R. Kneubehl, Megan B. Vogt, Jennifer L. Spencer Clinton, Rebecca R. Rico-Hesse

**Affiliations:** 1 Center for Comparative Medicine, Baylor College of Medicine, Houston, Texas, United States of America; 2 Department of Molecular Virology and Microbiology, Baylor College of Medicine, Houston, Texas, United States of America; 3 Section of Infectious Disease, Department of Internal Medicine, Baylor College of Medicine, Houston, Texas, United States of America; 4 Section of Pediatric Infectious Diseases, Department of Pediatrics, Baylor College of Medicine, Houston, Texas, United States of America; 5 Integrative Molecular and Biomedical Sciences Graduate Program, Baylor College of Medicine, Houston, Texas, United States of America; NIAID Integrated Research Facility, UNITED STATES

## Abstract

Chikungunya virus (CHIKV) is an emerging, mosquito-borne alphavirus responsible for acute to chronic arthralgias and neuropathies. Although it originated in central Africa, recent reports of disease have come from many parts of the world, including the Americas. While limiting human CHIKV cases through mosquito control has been used, it has not been entirely successful. There are currently no licensed vaccines or treatments specific for CHIKV disease, thus more work is needed to develop effective countermeasures. Current animal research on CHIKV is often not representative of human disease. Most models use CHIKV needle inoculation via unnatural routes to create immediate viremia and localized clinical signs; these methods neglect the natural route of transmission (the mosquito vector bite) and the associated human immune response. Since mosquito saliva has been shown to have a profound effect on viral pathogenesis, we evaluated a novel model of infection that included the natural vector, *Aedes* species mosquitoes, transmitting CHIKV to mice containing components of the human immune system. Humanized mice infected by 3–6 mosquito bites showed signs of systemic infection, with demonstrable viremia (by qRT-PCR and immunofluorescent antibody assay), mild to moderate clinical signs (by observation, histology, and immunohistochemistry), and immune responses consistent with human infection (by flow cytometry and IgM ELISA). This model should give a better understanding of human CHIKV disease and allow for more realistic evaluations of mechanisms of pathogenesis, prophylaxis, and treatments.

## Author summary

In humans, CHIKV infections are mostly symptomatic, resulting in fever, viremia, and debilitating joint disease; viremia is present up to 13 days after the onset of fever and peaks at the onset of clinical signs. Previous studies in mice and nonhuman primates (NHPs) have often neglected the mosquito vector, leading to variable viremias and infection of tissues primarily at the sites of CHIKV injection (legs in mice, ankles or spleen/liver in NHPs). This is the first study to demonstrate systemic CHIKV infection caused by infected mosquito bites in humanized laboratory mice. Disease seen with this humanized mouse model mimics human clinical signs and symptoms, with signs of inflammation in muscle and joints due to viral replication. This humanized mouse model provides a basis for assessing human CHIKV pathogenesis, prophylaxis, and treatment, under natural conditions of infection.

## Introduction

Chikungunya virus (CHIKV) is an emerging alphavirus in the *Togaviridae* family and is transmitted by *Aedes* species mosquitoes (usually *Ae*. *aegypti* and *Ae*. *albopictus*). CHIKV was originally discovered in 1952 in Tanganyika (modern-day Tanzania), East Africa and has been causing outbreaks globally since the mid-2000s [1–6]. The largest recent epidemic occurred in the Americas in 2014–2015 with over 1.5 million suspected cases and approximately 200 deaths [7]. Symptoms of CHIKV infection include fever, headache, muscle ache, rash, and debilitating joint disease [8]. Although most patients recover from CHIKV without recurrent acute infections, 50–95% of individuals can develop chronic joint symptoms lasting for months after the initial acute infection [9–13]. Most CHIKV infections are not life threatening, but severe infection can occur in immunodeficient individuals, such as neonates (infected during or prior to birth) and older individuals with comorbidities (diabetes mellitus, liver disease, obesity, or hypertension) [14–19]. Neurologic sequelae characterized by encephalomyelitis, myeloneuropathies, and neuro-ocular disease (uveitis, retinitis, optic neuritis) can also occur, resulting in long-term impairment and disability of immunocompromised children and previously healthy adults [20–22]. Prevention of human CHIKV cases through mosquito control has been used in the United States (e.g. Florida); however, success with this method has not yet been documented [23]. Currently there are no licensed vaccines or treatments for CHIKV, although some are in development [24].

Knowledge gained from animal models of CHIKV infections could aid in development of CHIKV vaccines or treatments. The most commonly used animal models for CHIKV infection are various types of mice or nonhuman primates (NHPs) [25–34]. While research using mouse or NHP models has contributed to a greater understanding of CHIKV, they also have several limitations that include infection with variable, sometimes extremely high, doses of virus [35] and abnormal administration routes (intraperitoneal, intracranial, or intravenous injection) that do not mimic natural infection [31]. Visual swelling of joints and histological evaluation of bones, joints, and musculature are often used to determine whether a model is useful for studying CHIKV. However, even though the virus can replicate in non-human hosts, mice tend to be asymptomatic [36–38], with wide variability in clinical symptom presentations. While various mouse models have been used to test CHIKV antiviral therapies or inform immune responses, they are also severely limited when extrapolating to human disease. Details of natural, mosquito-borne transmission are poorly defined or completely ignored in most models of CHIKV infection, despite differences in host immune responses when infection occurs by virus injection or mosquito bite [39]. Furthermore, mosquito saliva alone enhances viral pathogenesis [40–43], and multiple models of arboviral infection that use the natural vector (or administer mosquito saliva with the virus) better recapitulate human disease as opposed to models that do not account for mosquito transmission [40,43–55]. Additionally, proboscis probing and saliva release as mosquitoes feed/transmit pathogens is very complex [56], making it difficult to replicate transmission without the use of the vector itself. This gap in understanding the mechanics of virus delivery, immunology, and pathogenesis, illustrates the need for a novel model of CHIKV infection.

The humanized mouse model is a model in which severely immunocompromised mice are engrafted with human hematopoietic stem cells and/or lymphoid tissues. Consequently, engrafted mice develop elements of the human immune system, thus making them an extremely promising platform for studying arboviral infections. Historically, humanized mice have been useful for studying human infections difficult to replicate in wild-type mice, such as HIV and hepatitis C [57–59]. The humanized NSG strain (NOD.Cg-*Prkdc*^*scid*^ *Il2rg*^*tm1Wjl*^/SzJ), which develops human B cells, some T cells, dendritic cells, macrophages, monocytes, and natural killer cells, has been used by us to model dengue virus infection [54] and to evaluate the human immune response to mosquito saliva itself [43]. Here we investigated whether the humanized NSG (hu-NSG) mouse strain could serve as an effective model of CHIKV infection and pathogenesis.

Initially, we injected a reference strain of CHIKV (37997) intradermally into humanized NSG (hu-NSG) mice, to assess infection and compare this to other mouse models reported previously; we hypothesized that infection would be limited to the sites of injection, and that clinical signs would be minimal. Subsequently, we infected hu-NSG mice with the same strain of CHIKV through mosquito bites and compared infection to that of the injection model; we hypothesized that the mice would have a more systemic infection due to mosquito bite delivery, and have immune responses similar to those seen in humans. Here we show that hu-NSG mice infected with CHIKV by mosquito bite have some clinical signs of CHIKV human disease, and evidence of systemic infection, resulting in a more relevant model of human infection than reported in other mouse studies. Further refinement of this model (e.g. long-term studies, further characterization of lesions, using other viral genotypes) could facilitate the study of CHIKV pathogenicity, allowing for development of more relevant CHIKV therapies than are currently available.

## Materials and methods

### Ethics statement

All research performed was approved by the BCM institutional animal care and use committee (protocol #AN-6802) and complied with references from the Guide for the Care and Use of Laboratory Animals [60].

### Virus and cell preparation

Vero clone E6 (Vero) cells (CRL-1586, American Type Culture Collection (ATCC)) were maintained in Dulbecco’s Modified Eagle’s Medium (DMEM, ThermoFisher), containing 2% fetal bovine serum (FBS, Atlanta Biologicals) and penicillin/streptomycin (100U/mL). Vero cells were cultured at 37°C in 5% CO_2_. *Ae*. *albopictus* C6/36 cells (CRL-1660, ATCC) were maintained in Minimal Essential Media (MEM, ThermoFisher) with 10% or 2% FBS, 1% L-glutamine/penicillin/streptomycin (Sigma), 1× non-essential amino acids (Sigma), and 1% sodium pyruvate (Sigma). C6/36 cells were cultured at 28°C in 5% CO_2_.

CHIKV reference strain 37997 (West African genotype) was isolated from an *Ae*. *furcifer* mosquito in Kedougou, Senegal in 1983 (Genbank accession #AY726732), and was obtained from the Yale Arbovirus Research Unit [61]. The virus was passaged once in AP61 mosquito cells followed by one passage in Vero cells. Upon receipt, we passaged the virus a third time in either C6/36 or Vero cells. The supernatant was harvested from the CHIKV-infected cells after 3 days, cellular debris was removed with centrifugation (10,000 ×g for 10 minutes), and the supernatant was supplemented with 30% gelatin (C6/36) or FBS (Vero) before storage at -70°C. Virus passaged on C6/36 cells was used to inject hu-NSG mice subcutaneously, while virus passaged in Vero cells was used to inoculate mosquitoes. All work with infectious virus was performed under biosafety level 3 (BSL-3) laboratory conditions.

To determine viral titer, indirect immunofluorescent assays (IFAs) were performed on 10-fold serial dilutions of CHIKV 37997 stock. Vero cells at 10^5^ cells/well were incubated in 8-well LabTek II chamber slides (Nalge Nunc International). The next day, cells were inoculated with virus serial dilutions (from thawed stock, not re-frozen) for 1 hour at 37°C. After washing virus from the cells, slides were incubated at 37°C for 72 hours, then fixed in acetone. Slides were blocked in phosphate buffered saline (PBS, HyClone) with 2% FBS, then incubated with a primary mouse monoclonal antibody to CHIKV E2 glycoprotein (1:50 CHK-48, #NR-44002, BEI Resources) at 37°C for 2 hours. After washing, slides were incubated with a FITC-Goat α-Mouse IgG secondary antibody (1:100, Invitrogen) in a humidified chamber at 37°C for 1 hour. After incubation, slides were washed with PBS and mounted using ProLong Gold antifade reagent with DAPI (Life Technologies). Similar to what is done with a TCID50, virus titer was determined based on the lack of immunofluorescence as the cutoff.

### Mouse maintenance and engraftment

Non-obese diabetic, severe combined immune deficient, interleukin-2-receptor γ^-/-^ mice (NOD.Cg-*Prkdc*^*scid*^ *Il2rg*^*tm1Wjl*^/SzJ, The Jackson Laboratory), more commonly referred to as NSG mice, were maintained in a colony at an AAALAC-accredited facility under specific pathogen-free conditions. Hu-NSG mice were prepared as previously described [54]. In brief, human cord blood from anonymous donors (MD Anderson Cancer Center, Houston, TX) was obtained for the isolation and purification of CD34^+^ cells (CD34-positive selection kit II, Stem Cell Technologies). Approximately 3×10^5^ CD34^+^ cells were injected intrahepatically into sub-lethally irradiated (1cG) NSG pups 24 to 48 hours after birth. Mouse engraftment was confirmed at approximately 8 weeks of age through flow cytometry analysis of peripheral blood stained for human CD45 (APC-conjugated anti-human CD45, BD Biosciences) and mouse CD45 (FITC-conjugated anti-mouse CD45, BD Biosciences). Samples were analyzed on a Cantoll analyzer (BD Biosciences) in the Cytometry and Cell Sorting core facility. Data were collected using FACSDiva software (BD) and analyzed using FlowJo (v10.2, FlowJo, LLC). Mice were considered engrafted and suitable for study when blood contained 20–80% human CD45^+^ cells, as is consistent with standard humanized mouse models [44,54,62–64]. Though every attempt was made to have equal numbers of male and female mice available for experimentation, this was not always possible due to the size and sex of litters born, the percent of individual engraftment, and the timing of the study being performed.

### Mosquito rearing

*Ae*. *aegypti*, substrain Rockefeller, mosquito eggs (MRA-734, BEI Resources) were propagated as previously described [43]. In brief, eggs were flooded with distilled water in glass-covered plastic containers for hatching. Larvae were fed a mixture of ground rabbit pellets, yeast, and liver powder (1:1:1, w/w). Pupae were collected into hatching cups and hatched mosquitoes were maintained in a mesh cage in an insectary (26–28°C, 70–80% humidity) and fed 10% sucrose (Sigma) *ad libitum*. Subsequent generations of eggs were obtained by providing mosquitoes with a blood meal from an anesthetized C57BL/6 mouse (Center for Comparative Medicine (CCM)). Eggs were collected on damp pieces of paper, dried and stored within the insectary until further hatchings were needed.

### Mosquito infection and virus detection

Female mosquitoes, ages 3–7 days post-eclosion, were collected in plastic containers with mesh tops and transferred to the BSL-3 facility. Mosquitoes were immobilized on ice and infected with approximately 34,500 infectious units of CHIKV strain 37997 via intrathoracic microinjection as previously described [65]. Mosquitoes recovered from injection in plastic holding cups at a density of approximately 10 per cage, and were reared in humidified incubators (70–80% humidity, 29–31°C) with *ad libitum* sucrose.

Starting at 5 days post-injection, a single mosquito was selected daily for detection of CHIKV via IFA. The head was squashed into one well of a 12-well slide and a second, non-infected mosquito head was squashed as a negative control. Heads were fixed in acetone, blocked in PBS + 5% FBS, and stained with primary CHIKV antibodies (1:100 CHK-263, NR-44003 and/or 1:50 CHK-48, NR-44002, BEI Resources) and secondary detection antibody (1:200 anti-mouse FITC, Invitrogen). Slides were read on a fluorescence microscope to detect CHIKV E protein within the squashed mosquito head/salivary glands. Typically, a positive result was obtained by 9 days post-intrathoracic injection.

### Mouse infection via needle inoculation

To ensure hu-NSG mice could develop CHIKV infection, a pilot study was performed using needle inoculation. Engrafted mice (4–9 months of age) were transferred to the BSL-3 animal facility for acclimation at least 24 hours before initiation of experiments. Specific information on each mouse can be found in S1 Table. The lower abdomen was shaved at the time of transfer for future erythema readings. Animals were maintained 3 to a cage (Techniplast) with nesting enrichment, wireless low-profile running wheels (Med Associates, Inc.), *ad libitum* food, and sterile drinking water provided via water bottles.

Prior to injection, mice were anesthetized using 2–4% isoflurane with 2 liter/minute oxygen via nose cone. Stocks of CHIKV 37997 were diluted in complete MEM with 2% FBS. Each rear footpad was injected intradermally with 50 μl of virus preparation. In total, each mouse received 100 μl containing 11.8 log_10_ genome equivalents (approximately 2.3×10^8^ TCID50) of CHIKV strain 37997.

### Mouse infection via mosquito bite

Hu-NSG mice were transferred to the BSL-3 facility on the same day a positive IFA from mosquitoes was detected. On the day of infection, animals were sedated with approximately 100 mg/kg of ketamine and 5 mg/kg xylazine in a single intraperitoneal dose. Mosquito cups were placed mesh-side-down on both footpads of sedated mice to allow for mosquito feeding and engorgement, with 3–5 total mosquito bites per mouse. Feeding was confirmed through visualization of blood within the abdomen of the mosquitoes (engorgement). Exact number of mosquito bites, as well as information on each mouse, can be found in S2 Table. Animals in CHIKV groups received bites from mosquitoes that tested IFA positive after biting, and control animals received bites from non-injected mosquitoes. Mosquitoes were euthanized by placing the cups in a -20°C freezer for a minimum of 24 hours. Mouse weights were recorded while animals were sedated, and a 2 cm^2^ spot was shaved in the right axillary/upper abdominal region for erythema measurements.

### Mouse clinical signs and sampling

Animals were monitored daily for signs of pain, distress (hunched posture, ruffled coat, and grimace as previously defined [66]), and altered mobility (paresis/paralysis). Alternating groups of mice were sedated every 2–4 days for measurement of weight, rectal temperature (Physitemp Instruments), erythema (DSMII ColorMeter, Cortex Technology), and collection of 10 μl of retro-orbital blood. Blood was spun at 200 ×g for 5 minutes, and serum was collected and stored in TRIzol LS (Ambion) for subsequent viral RNA detection.

On designated endpoint dates, or as needed based on illness (moribund animal, inability to move to food or water, weight loss >20%), animals were euthanized via isoflurane overdose, and terminal blood was collected via cardiac puncture. Blood was placed into heparinized and non-heparinized containers for further processing. Non-heparinized blood was allowed to clot and spun for serum as previously described. Heparinized blood was lysed with 1x RBC lysis buffer (eBioscience) and centrifuged at 200 ×g for 5 minutes. Supernatant was removed, and the blood cell pellet re-suspended in PBS + 2% FBS. Skin from the footpads of both hind feet was removed and digested in 5 mg/mL collagenase (Worthington Biochemical Corporation). The skin was minced into smaller pieces and incubated for 1 hour at 37°C. After incubation, the samples were filtered through 40 μm filters (Falcon) and centrifuged at 200 ×g for 5 minutes. Supernatant was removed, and the skin cell pellet re-suspended in PBS + 2% FBS. Additional tissues collected postmortem included the left hind leg, right sciatic nerve, right femur, lungs, spleen, liver, and head/spinal cord. The bone marrow from one femur was flushed out of the marrow cavity with PBS + 2% FBS and filtered through a 40 μm filter. A sample of bone marrow suspension was stored in TRIzol LS for viral RNA isolation. Remaining sample was centrifuged at 200 ×g for 5 minutes. Supernatant was removed, and the bone marrow cell pellet re-suspended in PBS + 2% FBS. The spleen was collected and sectioned into thirds. Approximately 2/3 of the spleen was crushed between the frosted ends of a microscope slide and washed into a petri dish with PBS + 2% FBS. Spleen contents and PBS wash were filtered through a 40 μm filter. Remaining liquid was centrifuged at 200 ×g for 5 minutes. Supernatant was removed, and the splenocyte pellet re-suspended in PBS + 2% FBS. One lobe of the lung was macerated into a microcentrifuge tube using a sterile, plastic pestle then re-suspended in TRIzol for viral RNA isolation. All remaining collected tissues were fixed in 10% neutral buffered formalin.

### Tissue flow cytometry

Cells collected from blood, bone marrow, skin, and spleen were transferred to 96 well plates and incubated with antibodies against extracellular targets (S3 Table). All antibody incubations were performed for 30 minutes at 4°C. After staining, cells were fixed overnight in a 4% paraformaldehyde fixation buffer (Cytofix/Cytoperm, BD Biosciences). Fixed cells were washed with PBS + 2% FBS, re-suspended, and stored at 4°C until analysis. Samples were analyzed in the CCSC facility on the LSRII Fortessa (BD) using the High Throughput Sampler module. Data were collected using FACSDiva software and analyzed using FlowJo.

### Viral RNA extraction and qRT-PCR

All TRIzol-treated samples were stored at -70°C until use. Samples were thawed on ice and processed in accordance with manufacturer instructions using chloroform, 100% isopropyl alcohol, and 75% ethanol with the following modification: RNA was precipitated with 1 μl GlycoBlue (ThermoFisher). Precipitated RNA was re-suspended in 50 μl DEPC-treated water (Ambion). All samples were frozen and thawed on ice prior to running qRT-PCR.

Viral RNA was quantified by qRT-PCR in technical triplicates. A final reaction volume of 20 μl was used containing 10 μl serum or other sample RNA, 6.25 μl 4× TaqMan Fast Virus 1-Step Master Mix (Applied Biosystems), 9 pmol reverse primer, 9 pmol forward primer, 10 μM probe, and 1.75 μl DEPC water (Ambion). Primers and probe (S4 Table) were adapted from Pastorino and colleagues [67] who reported a sensitivity of 27 RNA copies and 1.2×10^−2^ infectious doses per reaction. Amplification was performed in a StepOnePlus instrument (Applied Biosystems) under the following cycling conditions: 1 cycle at 50°C for 20 minutes, 1 cycle at 95°C for 2 minutes, 45 cycles at 95°C for 5 seconds, and 60°C for 1 minute. CHIKV genome copies were determined by using a standard curve derived from a 10-fold dilution series of CHIKV RNA from passage 3 stock virus quantified by spectrophotometry.

### Live virus detection in serum

To confirm the presence of viral antigen in mouse serum, IFA was performed using serum from CHIKV-infected hu-NSG mice frozen at -20°C and infection of Vero cells. Positive controls consisted of frozen stock virus and negative controls consisted of DMEM media. IFAs were performed in the same manner as described above for determining viral titer; however, serum or control samples were used in each well as opposed to viral dilutions. Neutralizing antibody titers were not determined here due to a lack of sufficient quantities of mouse serum.

### Detection of human CHIKV antibodies by ELISA

A qualitative Human IgM ELISA Kit (Human Anti-Chikungunya Virus IgM ELISA Kit, Abcam) was used per the manufacturer’s instructions. In brief, mouse serum was incubated in pre-coated wells for 1 hour at 37°C. After thorough washing, CHIKV-specific antigen was applied to wells and incubated for 30 minutes at room temperature. This process was repeated with a CHIKV-specific antibody, followed by streptavidin conjugate, as directed. After washing, a 3,3′,5,5′-Tetramethylbenzidine substrate solution was added for 15 minutes at room temperature, followed by a stop solution. The ELISA plate was read within 30 minutes of substrate addition, using 450nm absorbance on an electronic plate reader (Fisher Scientific). Samples were considered positive if the absorbance value was greater than 10% over the cut-off value and negative if the absorbance value was less than 10% under the cut-off value. Samples that were neither positive nor negative were deemed inconclusive/equivocal.

### Histology and immunohistochemistry of tissues

All formalin-fixed tissues were submitted to the CCM Histology Core for histological fixation in paraffin. Bony tissues were decalcified in sufficient quantities (enough to completely cover the tissue) of TBD-2 solution (Thermo Scientific) for 24 hours, then returned to 10% neutral buffered formalin. Histological analysis was performed on the proximal joints (stifle joint; sectioned vertically through the joint to include the marrow cavity and the joint itself), distal joints (tarsal, metatarsal, and phalangeal joints; sectioned vertically through the tarsal joint with phalanges attached), heart, lung, spleen, sciatic nerve, brain/head, spinal cord, and gastrocnemius muscle. All samples were stained with hematoxylin and eosin (H&E) and analyzed by a board-certified pathologist.

Muscle and tendons were further analyzed in the CCM Histology Core with immunohistochemistry (IHC). Samples were stained for CHIKV envelope-specific monoclonal antibody (1:75, CHK-263, BEI Resources) using protocols established by the laboratory. Briefly, paraffin-embedded tissues were cut into 3–4 μm sections and placed on slides. Slides were heated at 60°C for 1 hour then deparaffinized with xylene and ethanol followed by antigen retrieval solution EDTA (Biocare) for 30 minutes. Slides were then allowed to cool and sections were inactivated of endogenous peroxidase and then incubated with the monoclonal antibody for 1 hour at 4°C. Counterstaining was performed with hematoxylin followed by mounting with permanent media. All IHC stained slides were analyzed by a board-certified pathologist.

### Statistics

Statistical analysis of mouse parameters (temperature, erythema, weight, wheel running) and flow cytometry data was performed using GraphPad Prism 7 software. Unpaired individual t-tests were used to determine significance between control and infected groups at each timepoint for the temperature, erythema, flow cytometry, and weight experiments. All error bars indicate standard error of the mean (SEM) as a measure of variance. Statistical significance is denoted by p values, using the following abbreviations: n.s. = not significant, *p<0.05, **p<0.01, ***p<0.005, ****p<0.001. A sixth order polynomial non-linear regression was used to assess differences in wheel running between control and infected mice groups. For flow cytometry, outliers were removed via ROUT analysis, and statistical significance was assessed via multiple comparison t-test using the Holm-Sidak correction.

## Results

### Hu-NSG mice infected via mosquito bite, but not by needle inoculation, demonstrate signs of CHIKV infection

**Fig 1:**
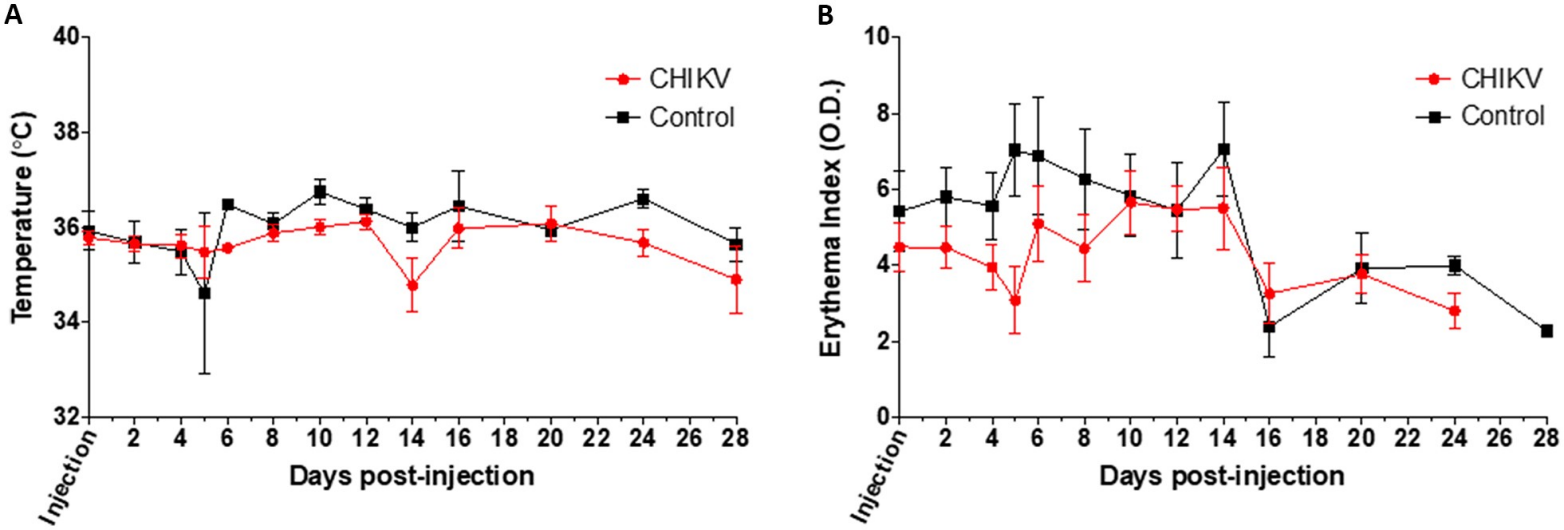
Clinical signs of CHIKV infection in Hu-NSG mice after needle inoculation into the rear footpad. (**A**) Temperature and (**B**) erythema index of CHIKV-injected mice as compared to control mice injected with saline were assessed as a measure of disease up to 22 days post-infection. CHIKV-injected mouse data are shown in red, while uninfected control mice data are indicated in black. Unpaired t-tests were used to determine statistical significance between CHIKV-injected and uninfected mice at each time point; however, no significant differences were seen when comparing CHIKV-injected mice with saline-injected mice. Mice per group: n≥7.

To determine whether hu-NSG mice develop illness following CHIKV inoculation, temperature, erythema, weight, and wheel running data were recorded. Thirty hu-NSG mice were inoculated with an intradermal injection into the footpad with CHIKV 37997 and 13 hu-NSG mice were injected intradermally in the footpad with media alone as mock-infected controls. All non-control mice were confirmed to be infected via qRT-PCR. There were no significant differences detected in temperature or erythema between control and CHIKV needle inoculated groups at any of the time points for animals infected via needle inoculation (Fig 1). Additionally, histological analysis of stifle joints did not reveal any evidence of arthritis, myositis, or tenosynovitis. Therefore, we concluded that hu-NSG mice infected via needle inoculation do not develop significant clinical signs following CHIKV infection.

**Fig 2:**
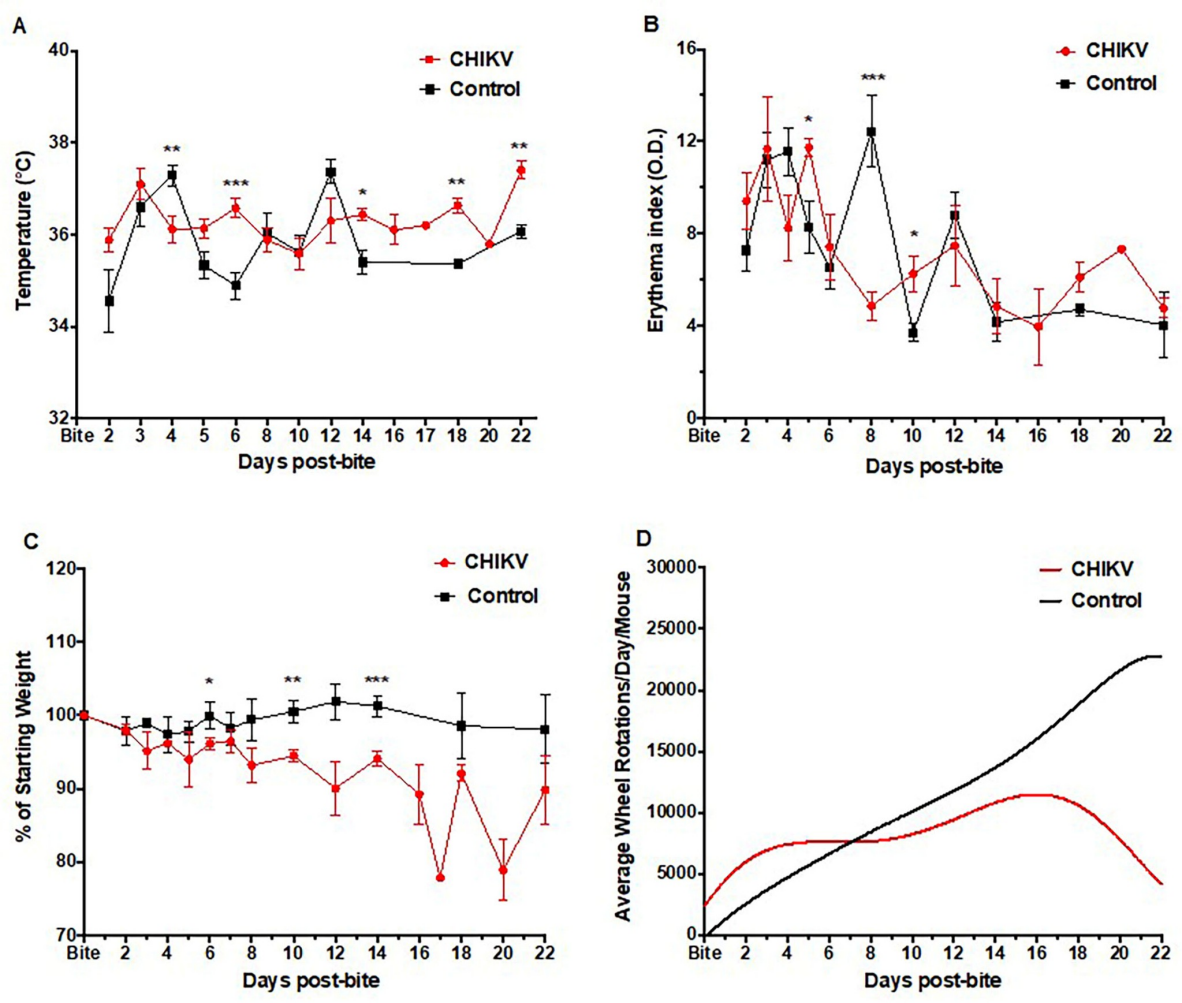
Clinical signs of CHIKV infection in Hu-NSG mice after natural transmission via mosquito bite. (**A**) Temperature, (**B**) erythema index, (**C**) weight change, and (**D**) activity levels (wheel rotations) of mosquito-bitten CHIKV-infected mice as compared to uninfected control mice bitten with non-infected mosquitoes were assessed as a measure of disease up to 22 days post-infection. CHIKV-infected mouse data are shown in red, while uninfected control mice data are indicated in black. Unpaired t-tests were used to determine statistical significance between CHIKV-infected and uninfected mice at each time point. Asterisks indicate significance differences (* p<0.05; ** p<0.01; ***p<0.005). Error bars indicate SEM. Wheel rotation curves were generated by a sixth order polynomial. Mice per group: n≥9.

To demonstrate that natural infection via mosquito bite would produce a better model than needle inoculation, we infected mosquitoes with CHIKV 37997 for hu-NSG mice studies. Eighteen hu-NSG mice received 3–6 bites from CHIKV-infected mosquitoes, while fifteen hu-NSG mice received 3–6 bites from non-infected mosquitoes. Mosquito bitten mice were assessed for signs of CHIKV illness by measuring temperature, weight, erythema, and wheel running activity. Unlike with needle inoculation, our data for mosquito bitten hu-NSG shows significant differences when comparing mice bitten by CHIKV-infected mosquitoes to control mice bitten by non-infected mosquitoes. Temperature of the mice bitten by CHIKV-infected mosquitoes showed significant elevation at days 6, 14, 18, and 22 after mosquito bite (Fig 2A). A significant decrease in temperature was noted at day 4. However, one mouse required early euthanasia at this time point (euthanasia performed after temperature data recorded). The fluctuation between elevated and normal temperatures is similar to the biphasic fluctuation of fever and associated clinical signs seen with human CHIKV presentations [68–70].

Mice bitten by CHIKV-infected mosquitoes showed significant increases in erythema readings on days 5 and 10, with a significant decrease noted on day 8 (Fig 2B), as compared to control mice bitten by uninfected mosquitoes. Presence of a rash can be variable among human disease [68,69,71–73], however when present it is concomitant with fever spikes [74].

Each mouse was normalized to its starting weight to ensure accurate tracking of weight changes. Weight loss was seen in all mosquito CHIKV-infected animals with significantly greater losses noted on days 6, 10, and 14 (Fig 2C), compared with uninfected mosquito-bitten control mice. Initial weight loss was noted in control mice after ketamine sedation, however all control mice returned to or exceeded their baseline weight by 6 days post-sedation as is consistent with the literature on mouse weight loss and recovery post-sedation [75]. The mild weight loss/gain noted at alternating timepoints is a by-product of individuals within the sub-group being weighed, thus creating a slight oscillation between timepoints.

Wheel running data (revolutions per day) were analyzed and averaged, based on the number of animals present in the cage. Although no statistically significant differences could be observed due to individual animal and cage variability, the overall trend of wheel running indicates a decrease in wheel activity in mosquito CHIKV-infected mice (Fig 2D).

### Hu-NSG mice bitten by infected mosquitoes have higher circulating viral RNA

To determine whether CHIKV-infected hu-NSG mice were viremic, retro-orbital blood was collected every 2–4 days and analyzed for the presence of CHIKV RNA using qRT-PCR. The viral RNA curve was created by combining results from all CHIKV-infected animals sampled at all of the time points in the study. For mosquito-bitten mice there is no viremia input level at day 0 due to the unknown quantity of virus deposited by mosquito bites (range 3–5 bites), however based on previous published data on amounts of CHIKV secreted in mosquito saliva, we estimated there to be 10^2^−10^4^ CHIKV virions injected per mosquito bite [76–79].

**Fig 3:**
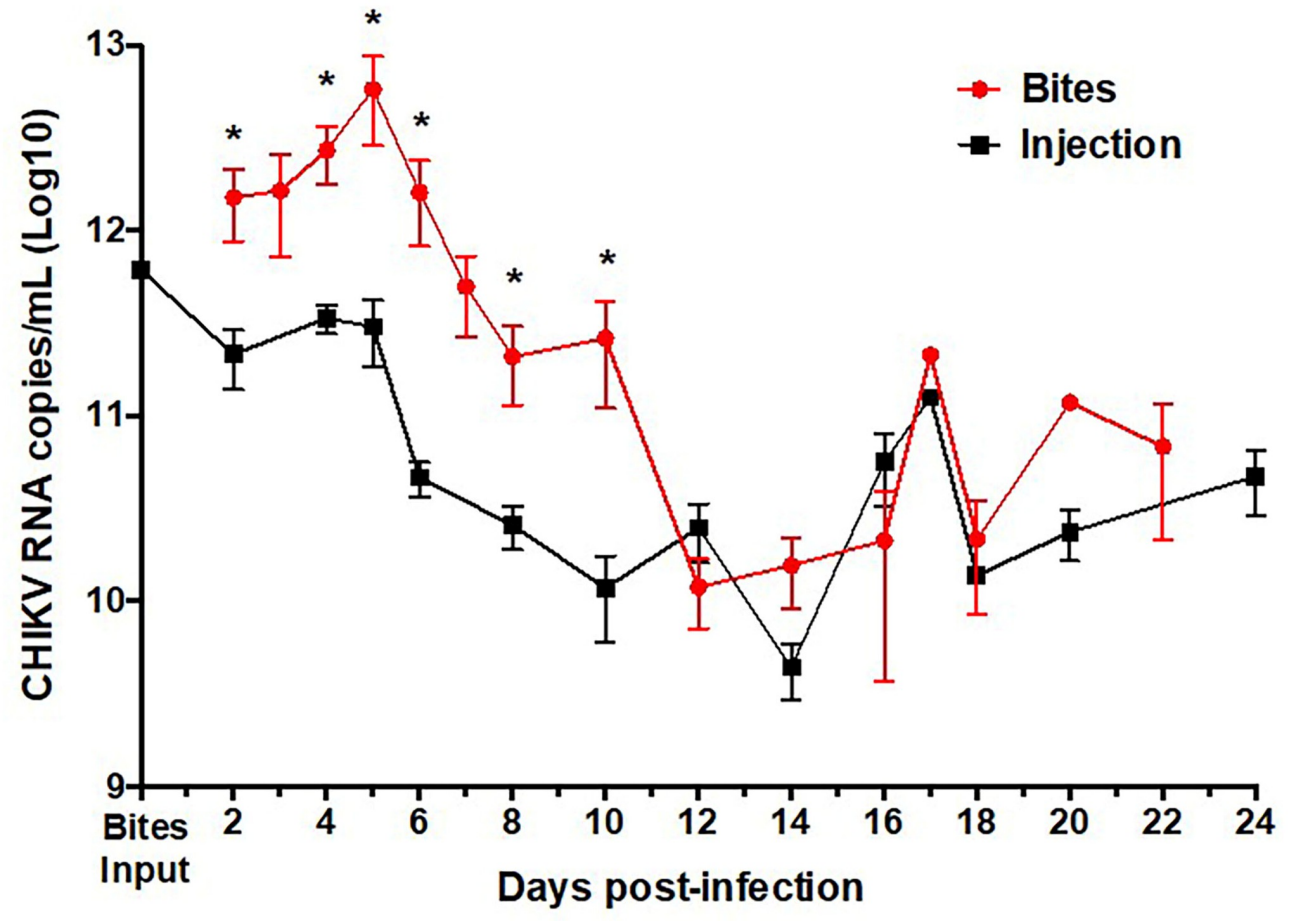
Virus in blood of CHIKV-infected Hu-NSG mice with mosquito bite or needle injection. CHIKV RNA copies/mL obtained from sera of humanized mice infected by mosquito bite (red) or subcutaneous injection (black) quantified by qRT-PCR. Unpaired t-tests were used to determine statistical significance between infection route at each time point. Asterisks indicate significance differences (* p<0.05; ** p<0.01; ***p<0.005). Error bars indicate SEM. Mice per group: n≥6.

For mice infected via needle inoculation our results showed the presence of viral RNA up to 28 days post-injection with the highest concentration of viral RNA detected at 5 days post-infection (approximately 10^12^ RNA copies/mL) (Fig 3). This is consistent with human viremia data indicating that the viremia peaks at or near the onset of clinical illness [8,80]. Human viremia levels are reported to be as high as 10^10^ viral particles per mL of blood [80,81] for prolonged periods after infection. CHIKV RNA copies/mL of hu-NSG infected via injection meet this level with an average range of 9 log10–11 log10 RNA copies/mL.

For mosquito-inoculated mice, viremia also peaked 5 days after mosquito bite then gradually declined thereafter (Fig 3). Despite the unknown quantities of CHIKV injected by approximately 4 mosquito bites, viral RNA from mosquito bite was present at significantly higher levels at day 5, as compared to the injection model (approximately 10^13^ RNA copies/mL). Persistent viral RNA was seen in all animals at 28 days post-bite, which is consistent with human viremia levels. During human CHIKV infection, CHIKV RNA has been reported from 1 week after infection [80] up to 17 days after presentation of clinical signs (fever, rash, or arthralgia) [82]. CHIKV RNA copies/mL of hu-NSG infected via mosquito bite range from 10 log10 to 13 log10 RNA copies/mL, meeting or exceeding the 10^10^ viral particles per mL of blood [80,81] seen in humans.

These data indicate that infection via mosquito bite can lead to more robust viral replication *in vivo* than by the injection of virus alone. This enhancement of infection has been seen using dengue virus to infect the same type of hu-NSG mice, also bitten by 4 infected mosquitoes [44], as well as with other arboviral infections [40,45,46,48,55].

### Infectious CHIKV and dissemination are detected in mice bitten by CHIKV-infected mosquitoes

To determine whether CHIKV dissemination and systemic infection occurred in hu-NSG mice, several further analyses were performed. In addition to viral RNA in the peripheral blood, the presence of viral RNA was confirmed by qRT-PCR in tissues (bone marrow, lung, and liver) collected at euthanasia time points (Table 1). Viral RNA was detected in all infected animals, in addition to CHIKV RNA being present in multiple locations (Table 1, columns 2–4). Our data, as measured by IHC and RT-PCR in serum and tissues, is consistent with viral dissemination seen in humans who died and were confirmed by the Enhanced Fatal AFI Surveillance System to have a CHIKV infection [18]. As CHIKV disseminates in humans, detectable levels can be seen in the liver, muscles, joints, and lymphoid tissues (lymph nodes and the spleen) [83]. Additionally, previous research has demonstrated the transmission of CHIKV via the aerosol route or presence in the orinasal cavity and lungs [84,85], thus presence of virus in lung tissues of our hu-NSG would be expected. These data indicate that CHIKV dissemination occurred and caused additional sites of infection (the liver and the lungs).

**Table 1:** CHIKV is detected in various humanized mouse tissues after infection via mosquito bite.

Experimental group:	Bone marrow average RNAc (SEM)	Liver average RNAc (SEM)	Lung average RNAc (SEM)	ELISA IgM	Serum IFA Positive results
CHIKV 7 day bite	2.87E+11 (5.73E+10)	9.56E+09 (8.01E+09)	1.46E+09 (7.88E+08)	3/6	5/6
CHIKV 14 day bite	1.89E+10 (1.73E+10)	2.28E+04 (2.28E+04)	7.93E+11 (7.93E+11)	3/6	6/6
CHIKV 25 day bite	3.50E+10 (1.67E+10)	3.49E+06 (3.33E+06)	6.02E+08 (4.10E+08)	2/4	4/4

Presence of CHIKV RNA copies (RNAc) in bone marrow, livers, and lungs were detected via qRT-PCR. Infectious virus and CHIKV-specific IgM antibodies were evaluated in sera, and assessed by limiting dilution assay (Serum IFA) and CHIKV IgM ELISA kit, respectively. Control mice (n = 19) were CHIKV negative (or below limits of detection) in these assays.

Additionally, we investigated systemic CHIKV infection in hu-NSG mice by assessing the production of infectious CHIKV virions. Serum samples collected at euthanasia were used to infect Vero cells, and these serum-inoculated cells were visualized with IFA. After inoculating Vero cell monolayers with serum, a 72-hour incubation, and staining with CHIKV-specific antibodies, virus was detected in 15 of 16 terminal samples (Table 1, column 6). The only serum sample not containing infectious virus was collected at 7 days post-mosquito bites, from one mouse, and we hypothesize that viremia had likely not yet peaked in this animal, thus resulting in a negative IFA.

Another confirmation of systemic mouse exposure was done by assessing seroconversion. Detection of specific antibodies against CHIKV was performed on serum samples collected at euthanasia using a human ELISA kit specific for IgM. According to the kit instructions and cutoff points, approximately 50% of the CHIKV-mosquito bite-infected mouse samples were positive for IgM (Table 1, column 5 and S5 Table). None of the control samples showed a positive result for CHIKV IgM (Table 1, column 5 and S5 Table). One CHIKV bitten mouse had an equivocal result for day 7, but no excess serum was available to re-run the assay. In humans, IgM typically appears 6–8 days after clinical signs, and is present thereafter [86]. The detection of human IgM in only 50% of the mice, and an equivocal result on day 7 could be due to the fact that IgM is not fully present until the viremia begins to decline [87], or possibly because mice were humanized to varying degrees (see S2 Table). Positive results on the human-based ELISA further indicate viral exposure, and a human B-cell immune response to the infection. Taken together, these data suggest that CHIKV infection of hu-NSG mice by mosquito bite is able to produce systemic infection, infectious viremia, and IgM seroconversion, thus making this a model that can be useful in further studies of CHIKV infection and disease.

### Long-term CHIKV infection and pathology are detected in hu-NSG mice bitten by infected mosquitoes

**Fig 4:**
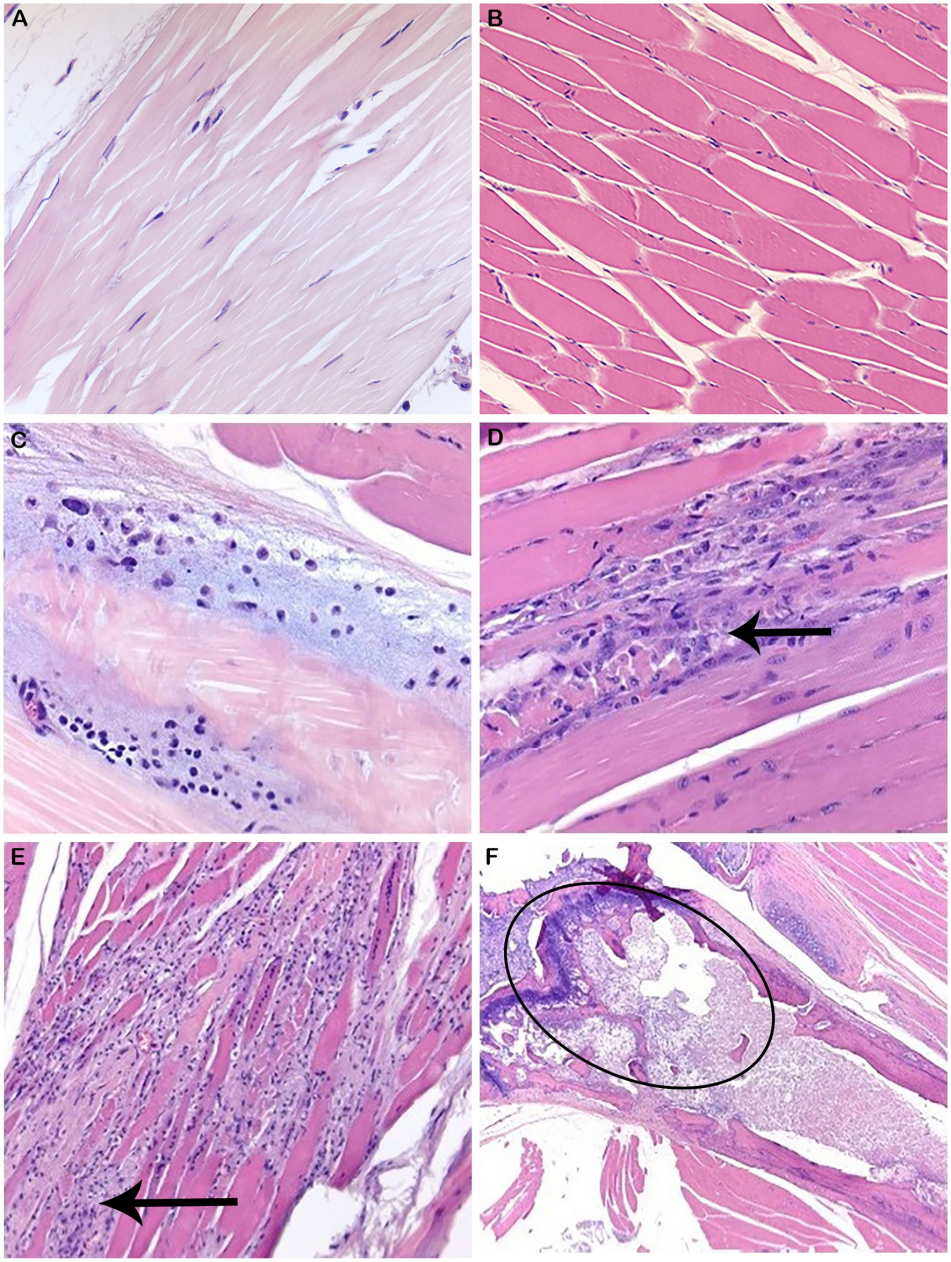
Histopathological lesions in CHIKV-infected Hu-NSG mouse joints and tissues. Histological staining of control and CHIKV-infected mouse tendon, muscle, and bone marrow from the region of the stifle. (**A**) Normal tendon (40X) from a control mouse. (**B**) Normal musculature (20X) from a control mouse. (**C**) Tendon histopathology showing tendonitis (Score = 1, 40X) in the tendons around the tarsus at 7 days post-infection via mosquito bite on the rear footpads. (**D**) Muscle histopathology indicating myositis of stifle musculature (black arrows) at day 14 post-infection (Score = 2, 40X) via mosquito bite of the rear footpads. (**E**) Muscle histopathology indicating myositis of paw/digit musculature (black arrows) at day 25 post-infection (Score = 3, 20X) via mosquito bite of the rear footpads. (**F**) Histopathology of bone marrow necrosis in the femur (black circle, 10X) at 7 days post-infection via mosquito bite of the rear footpads.

To determine whether disease seen in the mice was truly related to CHIKV infection, histopathology, and IHC for CHIKV viral proteins was performed. Histological analysis of stifle joints from both the CHIKV needle inoculation experiments and mosquito-bitten experiments were assessed by a board-certified pathologist. Joint histology from needle-inoculated animals did not reveal any evidence of arthritis, myositis, or tenosynovitis. However, mice bitten by infected mosquitoes resulted in histological lesions. Histological evaluation by a board-certified pathologist of soft tissue samples from mosquito bitten mice is summarized in Table 2. Representative photographs are shown in Fig 4. In summary, 50% of mice bitten by infected mosquitoes demonstrated CHIKV-related lesions on day 7, including bone marrow necrosis, myositis, or tendonitis. At 14 days post-bite, 66.7% of mice showed CHIKV-related lesions with synovitis or myositis. At day 25, 83.3% of mice exhibited CHIKV-related lesions such as bone marrow necrosis, myositis, or tendonitis. The characteristics of the joint and muscle inflammation were similar in all locations, with infiltration of mostly mononuclear cells and some neutrophils. Inflammation occurred most commonly in the skeletal muscle causing myositis, however occasionally inflammation was present around tendons and in the synovial membrane or surrounding soft tissue (tendonitis, synovitis). This is similar to the tenosynovitis described in the literature for human infections [69]. There was often overlap with inflammatory cells surrounding muscle and the adjacent tendons, thus making differentiation between synovitis, myositis, or tendonitis slightly artificial. Inflammatory lesions in the hind limbs of CHIKV-infected mice were present at all of the time points examined and increased in incidence and severity over time, as is reported in chronic human infections [68,88]. Bone marrow necrosis, previously reported in other CHIKV mouse models [89], was present in three CHIKV bitten mice on days 7 and 25 post-infection. Although bone marrow necrosis has not been directly seen in human CHIKV cases, virus-associated vasculitis, bone marrow edema, and progressive erosive arthritis have been reported in humans and other mouse models of CHIKV infection [21,89–95]. It is plausible that bone marrow necrosis could occur secondary to vasculitis or edema induced by CHIKV infection. Histological analysis of humans with severe CHIKV is often not performed, so it is possible that bone marrow necrosis is present and underreported. As the length of these experiment increased, the severity of disease as well as prominence and dispersal of CHIKV envelope protein also increased.

**Table 2:** Summary of histological scorings of Hu-NSG mouse joints and tissues in relation to animal and group information.

Time Point	Group	Mouse ID	Sex	Age	Engraftment (%)	# bites	Inflammation / Cellular Infiltrate			Granulomatosis			Periocular	Other
							Knee / Gastrocnemius	Tarsus	Paw	Spleen	Marrow	Liver	Phlebotomy lesions	
														
7 days	CHIKV Bite	955	F	8	53.5	6	0—WNL	0—WNL	0—WNL	2	3	0	Y	
7 days	CHIKV Bite	961	F	7	36.2	4	0—WNL	0—WNL	0—WNL	2	2	0	Y	
7 days	CHIKV Bite	962	F	7	32.5	4	Bone Marrow Necrosis	0—WNL	0—WNL	3	4	4	Y	
7 days	CHIKV Bite	963	F	7	37.3	4	0—WNL	0—WNL	1—Myositis/tendonitis	2	2	0	Y	
7 days	CHIKV Bite	1000	F	6	33.8	4	0—WNL	0—WNL	0—WNL	3	2	0	Y	Spleen—ectopic bone formation
7 days	CHIKV Bite	1001	F	6	53.5	4	0—WNL	1—Tendonitis	0—WNL	0	3	0	Y	
7 days	Control Bite	930	F	7	29.1	4	0—WNL	0—WNL	0—WNL	0	3	0	N	
7 days	Control Bite	931	F	7	23.7	3	0—WNL	0—WNL	0—WNL	0	0	0	N	
7 days	Control Bite	932	F	7	33.3	5	0—WNL	0—WNL	0—WNL	2	4	0	N	
7 days	Control Bite	941	F	7	32.4	4	0—WNL	0—WNL	0—WNL	0	3	0	N	
7 days	Control Bite	947	F	6	45.8	4	0—WNL	0—WNL	0—WNL	2	2	0	N	
7 days	Control Bite	948	F	6	39.8	4	0—WNL	0—WNL	0—WNL	0	2	0	N	
														
14 days	CHIKV Bite	910	M	8	21.3	4	0—WNL	1—Synovitis	1—Myositis	0	0	0	Y	
14 days	CHIKV Bite	911	M	8	45	3	0—WNL	0—WNL	0—WNL	0	2	0	Y	
14 days	CHIKV Bite	952	F	5	63.8	4	2—Myositis	0—WNL	1—FB inflammation	0	0	0	Y	
14 days	CHIKV Bite	953	F	5	47.2	3	0—WNL	0—WNL	0—WNL	2	4	4	Y	
14 days	CHIKV Bite	954	F	5	59.3	2	2—Myositis	2—Myositis	0—WNL	0	0	0	Y	
14 days	CHIKV Bite	959	M	5	32.6	5	0—WNL	0—WNL	2—Myositis	0	2	0	Y	
14 days	Control Bite	933	F	7	25.6	4	0—WNL	0—WNL	0—WNL	0	0	0	N	2—otitis media
14 days	Control Bite	934	F	7	24.1	3	0—WNL	0—WNL	0—WNL	0	2	0	N	
14 days	Control Bite	935	F	7	37.4	3	0—WNL	0—WNL	0—WNL	2	2	2	N	
14 days	Control Bite	938	M	6	33.5	5	0—WNL	0—WNL	0—WNL	2	3	0	N	
14 days	Control Bite	939	M	6	36.4	4	0—WNL	0—WNL	1—FB inflammation	4	4	0	N	
14 days	Control Bite	942	M	6	24	4	0—WNL	0—WNL	0—WNL	0	3	0	N	
														
25 days	CHIKV Bite	956	M	6	41.4	5	0—WNL	0—WNL	3—Myositis	0	3	0	Y	
25 days	CHIKV Bite	957	M	6	32.7	4	0—WNL	1—Myositis	0—WNL	2	2	0	Y	
25 days	CHIKV Bite	958	M	6	41.7	5	0—WNL	2—Myositis	3—Myositis	2	4	0	Y	
25 days	CHIKV Bite	986	M	5	35.1	3	0—WNL	Bone Marrow Necrosis	1—FB inflammation	0	0	0	Y	
25 days	CHIKV Bite	988	M	5	32.5	3	0—WNL	0—WNL	0—WNL	0	0	0	Y	
25 days	CHIKV Bite	989	M	5	30	3	0—WNL	2—Myositis/tendonitis Bone Marrow Necrosis	0—WNL	0	0	0	Y	
25 days	Control Bite	960	M	6	48.5	5	0—WNL	0—WNL	0—WNL	2	4	0	N	
25 days	Control Bite	968	M	5	27.6	6	0—WNL	0—WNL	1—FB inflammation	4	3	0	N	
25 days	Control Bite	990	M	5	24	4	0—WNL	0—WNL	1—FB inflammation	0	0	0	N	

CHIKV-infected and control mouse tissues were processed and stained for histology. CHIKV lesions were scored on a scale from 0 to 4 (0 = None/WNL(Within Normal Limits); 1 = Minimal; 2 = Mild; 3 = Moderate; 4 = Severe). Highlighted findings are considered non-incidental.

**Fig 5:**
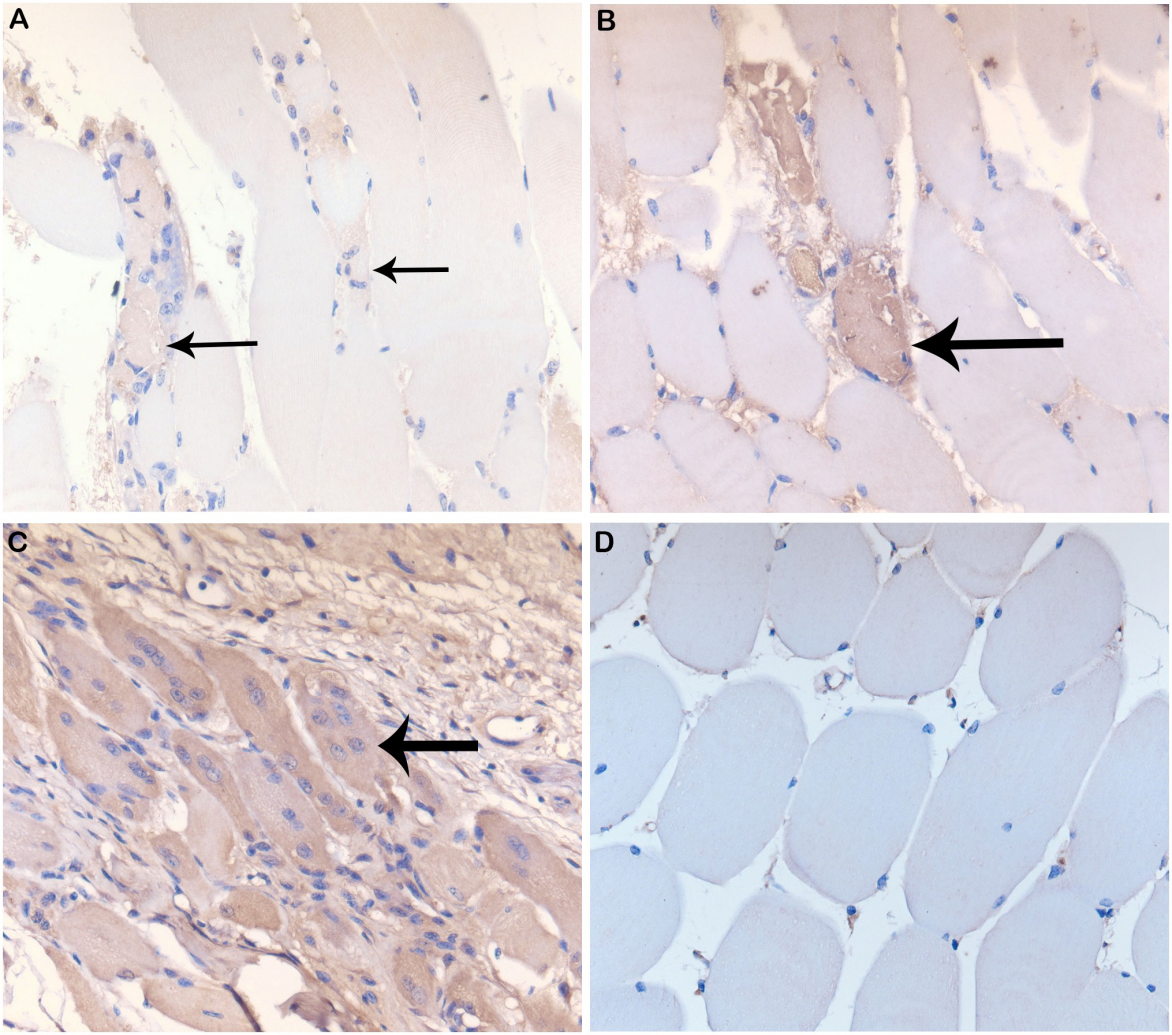
IHC of lesions in CHIKV-infected Hu-NSG mouse muscles. Immunohistochemistry (IHC) staining of CHIKV-infected mouse gastrocnemius muscle with CHK-263 antibodies. Positive staining (brown) represents CHIKV Env protein. (**A**) Control mouse with background IHC staining, 40X magnification. (**B**) Infected, degenerate muscle fibrils from minimal (score 1) myositis at day 7 post mosquito bite, 40X magnification. (**C**) Infected, degenerate muscle fibrils from mild (score 2) myositis at day 14 post mosquito bite, 40X magnification. (**D**). Infected muscle fibrils from moderate (score 3) myositis at day 21, 40X magnification.

In addition to the histological change indicative of CHIKV infection, IHC of myositis-affected muscles showed positive staining with CHK-263 antibody. This change was not seen among control animals (Fig 5). Based on our results, the clinical signs of CHIKV correspond to the location of CHIKV protein deposition. These data suggest that CHIKV infection in hu-NSG mice infected via mosquito bite, but not those infected via injection, induce long-term, pathological effects.

Incidental lesions found on histopathology (not pictured) also included: splenic granulomatosis, commonly found as an indicator of graft-versus-host-disease (GVHD) in humanized mice [96]; phlebotomy related inflammation in the periocular region and harderian glands of CHIKV infected mice [97]; foreign body inflammation of the dermis of the toes in 11% of CHIKV mice and 20% of control mice, likely related to small traumatic implantation sites that occur as the mice move or grasp objects; ectopic bone formation in the spleen of one CHIKV-infected mouse, an uncommon lesion that may represent colonization by human osteoblast progenitors present in the CD34 stem cell population or residual mouse osteoblastic cells; and otitis media of one control mouse, a common finding in immunodeficient mice most likely due to opportunistic bacterial infections.

### Hu-NSG mice bitten by infected mosquitoes show significant immunological changes

The immune system is integral in resolving CHIKV infection but may contribute to inflammation and arthritis in long-term infections. As such, we investigated the immune cell populations in our hu-NSG mice following mosquito bite infections, with a focus on dendritic cells (DCs), monocytes, macrophages (Mϕ), immune cells (T-cells, B-cells, natural killer (NK) cells, neutrophils), and T-cell subsets. In humans, the early stages of CHIKV infection are characterized by an antiviral response consisting of increased Mϕ, DC, and NK cells [98]. These cell types remain increased throughout the body for as long as viremia is present [99]. DCs are sequestered to the infection site (skin), thus causing perpetuation of the infection. In the chronic phase of CHIKV infection, macrophages are proposed to act as cellular reservoirs leading to persistence of the infection long after infection [100]. To determine whether our hu-NSG mice develop an immune response to CHIKV we used flow cytometry to detect human immune cells involved in the innate and adaptive immune responses. Immune cell populations from animals bitten by CHIKV-infected mosquitoes were compared to animals bitten by non-infected mosquitoes.

**Fig 6:**
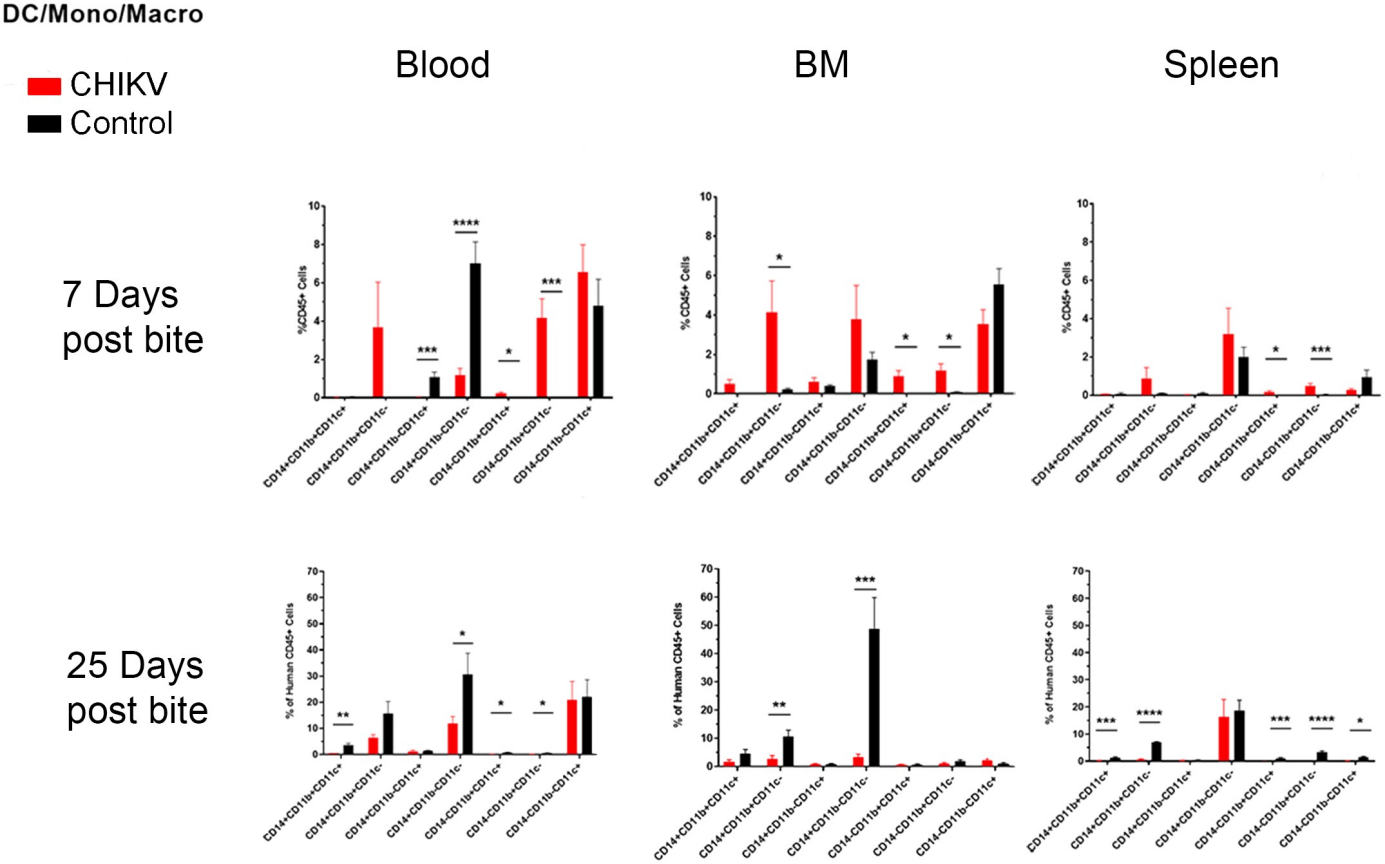
Summary of significant flow cytometry results (dendritic cells, monocytes, and macrophages) comparing Hu-NSG mice bitten by infected mosquitoes versus those bitten with control (uninfected) mosquitoes. Hu-NSG mice were infected with CHIKV via mosquito bite. At 7 and 25 days post-infection, mice were euthanized, and changes in immune cell populations in the blood, spleen, and bone marrow between infected and control mice were assessed via flow cytometry. Outliers were removed via ROUT analysis, and statistical significance was assessed via multiple comparison t-test using the Holm-Sidak correction. Immune components are outlined in the graphs above and summarized in table 5. Asterisks indicate significance differences (n.s. = not significant; * p<0.05; ** p<0.01; ***p<0.005).

At 7 days post-infected bite we observed a significant decrease in CD11c^+^ monocyte/Mϕ (CD14^-^CD11b^+^CD11c^+^) within the blood and a significant increase in CD11c^-^ monocytes (CD14^+^CD11b^+^CD11c^-^) in the bone marrow (as compared to control bitten mice) (Table 3 and Fig 6). By 25 days post-infection, there was a significant decrease in the overall monocyte/Mϕ population in the blood (CD14^+^CD11b^+^CD11c^+^), and a decrease in varying monocyte/Mϕ subpopulations throughout all tissues (CD14^+^CD11b^+^CD11c^-^, CD14^+^CD11b^-^CD11c^-^). Monocytes and Mϕ are critical to the CHIKV immune response, and an increase of these cells in the bone marrow at 7 days post-infection could indicate increased production to combat infection [98].

**Table 3:** Summary of significant flow cytometry results comparing Hu-NSG mice bitten by infected mosquitoes versus those bitten with control (uninfected) mosquitoes.

		Monocytes and Macrophages (Mϕ)					Dendritic cells (DC)			Immune Cells					T Cell Subsets	
		Overall Monocytes / Mϕ	CD11c+ Monocyte	CD11c- Monocyte	CD11c+ Monocytes / Mϕ	CD11c- Monocyte / Mϕ	MoDC	Myeloid DC	Unspecified DC	B Cells	Activated B Cells	T Cells	NK Cells	Neutrophils	DP T Cells	NK T Cells
**7 Days post infection**	**Blood**	n.s.	n.s.	n.s.	**↓** ***	**↓** ***	n.s.	↑*	↑***	n.s.	—	n.s.	n.s.	↑*	n.s.	n.s.
	**Spleen**	n.s.	n.s.	n.s.	n.s.	n.s.	n.s.	↑*	↑***	**↑** ***	—	n.s.	**↑** *	**↑** *	n.s.	n.s.
	**Bone Marrow**	**↑** *	n.s.	↑*	n.s.	n.s.	n.s.	↑*	↑*	n.s.	—	n.s.	n.s.	n.s.	↓*	↓**
**25 days post infection**	**Blood**	**↓** ***	**↓** **	n.s.	n.s.	**↓** *	n.s.	**↓** *	**↓** *	—	**↓** ***	**↑** ***	n.s.	**↓** *	n.s.	**↓** ***
	**Spleen**	n.s.	**↓** ***	**↓** ***	n.s.	n.s.	**↓** *	**↓** ***	**↓** ***	—	**↓** *	**↑** *	**↑** *	**↓** ***	n.s.	n.s.
	**Bone Marrow**	n.s.	**↓** **	n.s.	n.s.	**↓** ***	n.s.	n.s.	n.s.	—	**↓** *	n.s.	**↑** **	**↓** ***	n.s.	n.s.

Hu-NSG mice were infected with CHIKV via mosquito bite. At 7 and 25 days post-infection, mice were euthanized, and changes in immune cell populations in the blood, spleen, and bone marrow between infected and control mice were assessed via flow cytometry. Outliers were removed via ROUT analysis, and statistical significance was assessed via multiple comparison t-test using the Holm-Sidak correction. Immune components are summarized in this table. Arrows indicate whether a cell population was increased or decreased in mice bitten by infected mosquitoes compared with those bitten by uninfected mosquitoes. Asterisks indicate significance differences (n.s. = not significant;

* p<0.05;

** p<0.01;

***p<0.005).

For DC subsets, we observed significant increases in myeloid DCs (CD14^-^CD11b^+^CD11c^+^) and unspecified DCs (CD14^-^CD11b^+^CD11c^-^) in blood, spleen, and bone marrow at 7 days post-infection (Table 3 and Fig 6). At 25 days post-infection, we observed a decrease in myeloid DCs and unspecified DCs in the blood, and a decrease in monocyte-derived DCs (CD14^-^CD11b^-^CD11c^+^), myeloid DCs, and unspecified DCs in the spleen. The population of monocyte-derived DCs was decreased in the spleen but not in any other tissue tested. These data could indicate monocyte-derived DC migration to other parts of the body, including joints and muscles.

**Fig 7:**
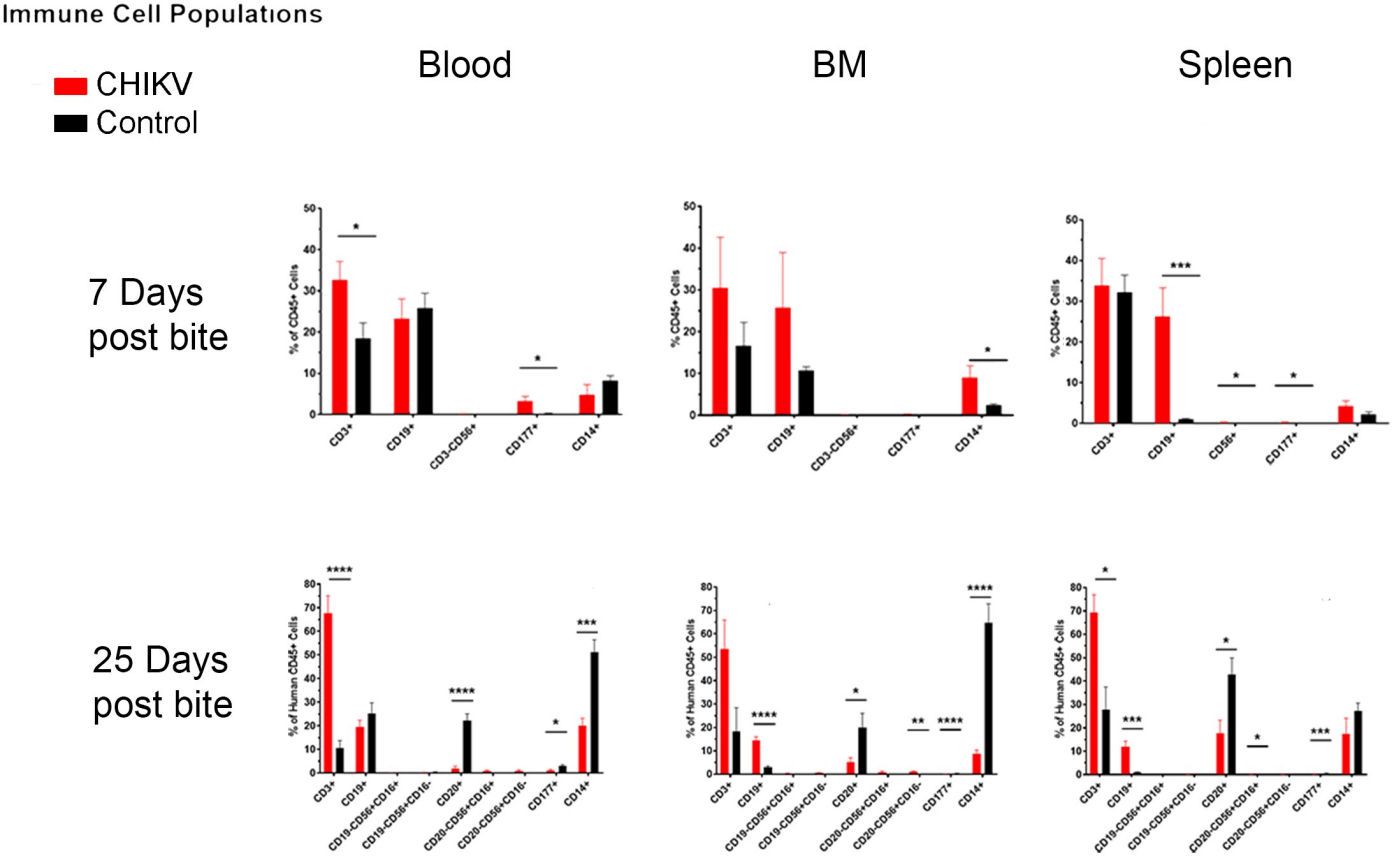
Summary of significant flow cytometry results (Immune cell populations: B cells, T cells, NK cells, and neutrophils) comparing Hu-NSG mice bitten by infected mosquitoes versus those bitten with control (uninfected) mosquitoes. Hu-NSG mice were infected with CHIKV via mosquito bite. At 7 and 25 days post-infection, mice were euthanized, and changes in immune cell populations in the blood, spleen, and bone marrow between infected and control mice were assessed via flow cytometry. Outliers were removed via ROUT analysis, and statistical significance was assessed via multiple comparison t-test using the Holm-Sidak correction. Immune components are outlined in the graphs above and summarized in table 5. Asterisks indicate significance differences (n.s. = not significant; * p<0.05; ** p<0.01; ***p<0.005).

Humoral and cellular responses, including B and T-cell responses, are very important in arthropod-borne viral infections, as they often lead to chronicity and an increased severity of disease [101]. Populations of human B and T-cells were also detected via flow cytometry to determine the relevance of humoral responses within our hu-NSG model. We observed an increase in total CD3^-^CD19^+^ B-cells (CD19^+^) in the spleen at 7 days post-infection and decreases in activated CD3^-^CD20^+^ B-cells (CD20^+^) in all tissues tested at 25 days post-infection (Fig 7 and Table 3). The decrease in activated B-cells at 25 days post-infection is similar to observations seen in human CHIKV infections [102]. Due to technical error, we do not have data for total B-cells at 25 days post-infection or activated B-cells at 7 days post-infection. During viral infections, B-cells with receptors specific for viral antigens proliferate and produce antibodies for neutralizing and clearing virus in lymph nodes and splenic germinal centers [103]. The increase of B-cells in the spleen at 7 days post-infection likely indicates that B-cells are responding to the infection. These data correlate with the production of CHIKV-specific IgM antibody detected in half of the infected mice beginning at 7 days post-infection (Table 1 and S5 Table).

**Fig 8:**
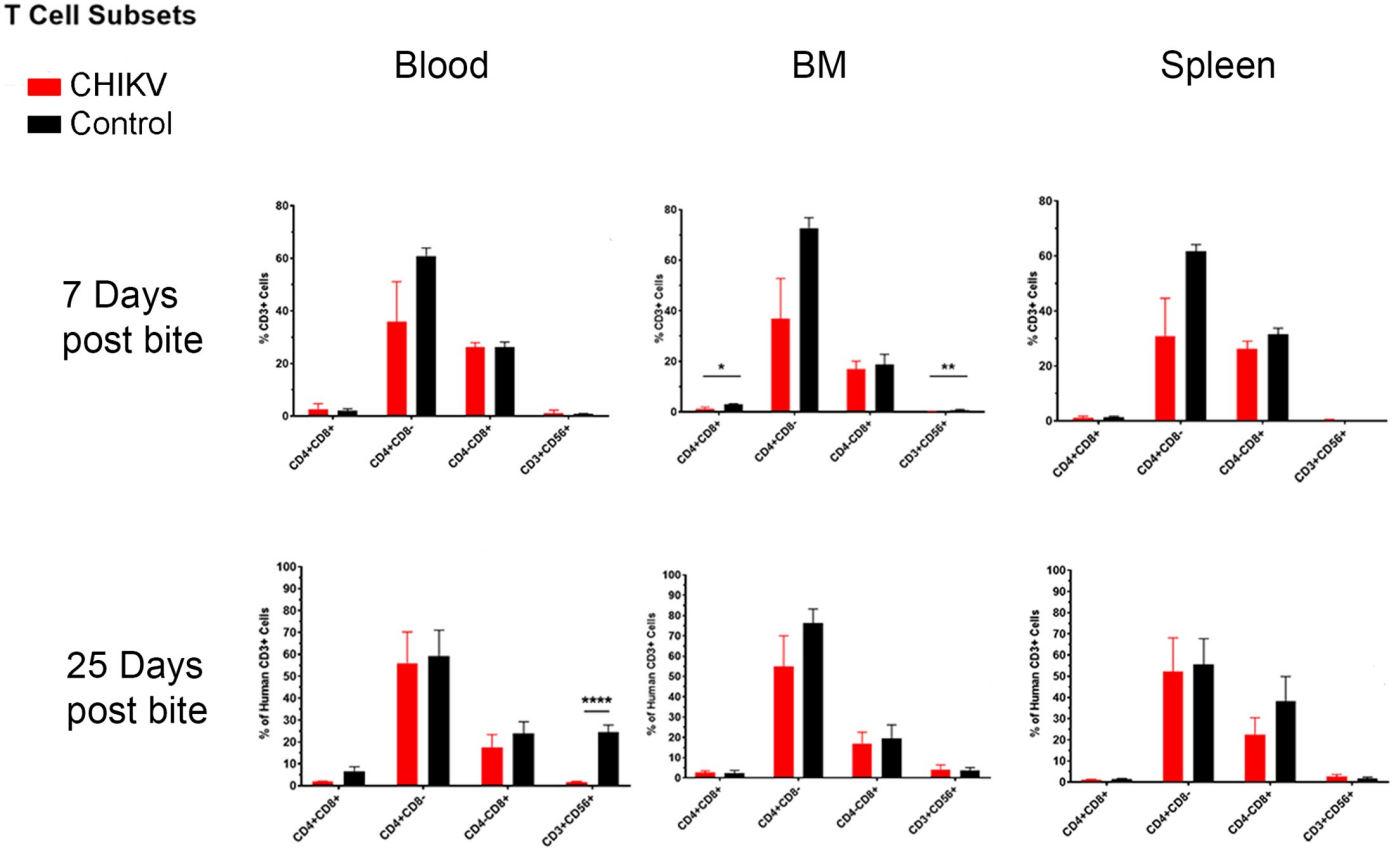
Summary of significant flow cytometry results (T cell subsets: Double positive T cells and NK T cells) comparing Hu-NSG mice bitten by infected mosquitoes versus those bitten with control (uninfected) mosquitoes. Hu-NSG mice were infected with CHIKV via mosquito bite. At 7 and 25 days post-infection, mice were euthanized, and changes in immune cell populations in the blood, spleen, and bone marrow between infected and control mice were assessed via flow cytometry. Outliers were removed via ROUT analysis, and statistical significance was assessed via multiple comparison t-test using the Holm-Sidak correction. Immune components are outlined in the graphs above and summarized in table 5. Asterisks indicate significance differences (n.s. = not significant; * p<0.05; ** p<0.01; ***p<0.005).

When examining total human T-cells (CD3^+^), we observed a significant increase in the blood beginning at 7 days post-infection, which became drastically increased by day 25 (Fig 7 and Table 3). However, there were no significant changes in CD4^+^ or CD8^+^ T-cells at any time point in any of the examined tissues (Fig 8 and Table 3). At 7 days post-infection we observed a significant decrease in CD4^+^CD8^+^ double positive (DP) T-cells and NK T-cells (CD3^+^CD56^+^) within the bone marrow (Fig 8 and Table 3). DP T-cells are a unique T-cell population that arise after thymic education when CD4^+^ or CD8^+^ single positive T-cells upregulate expression of the other cell surface receptor [104]. This usually occurs in the context of development but is also seen in response to various diseases [104]. The function of these DP T-cells is dependent on the original cell from which they are derived. For example, DP T-cells that arise from CD8^+^ T-cells have improved cytotoxic functions, while DP T-cells that arise from CD4^+^ T-cells are associated with inflammatory conditions, such as psoriasis and multiple sclerosis [104–111]. Mosquito saliva itself can also cause an increase in the amount of DP T-cells [43], indicating the importance of mosquito saliva in mosquito-borne diseases. We were unable to determine whether the DP T-cells found following CHIKV infection in our study were cytotoxic or pro-inflammatory.

At 7 days and 25 days post-infection, NK T-cells (CD3^+^CD56^+^) were significantly decreased in the bone marrow and blood, respectively (Fig 8 and Table 3). NK T-cells can kill infected cells in a manner similar to that used by cytotoxic T-cells. They also secrete cytokines (especially IL-4) that can stimulate anti-parasitic immune responses, which skews the immune response away from the anti-viral response [112–114]. The decreased NK T-cell populations observed post-CHIKV infection indicate a skewing of the immune response towards an anti-viral response. An analysis of cytokine production would be necessary to definitively characterize the function of the NK T-cells following CHIKV infection.

The last cell populations that we investigated were human NK cells (CD3^-^CD56^+^) and human neutrophils (CD3^-^CD177^+^). NK cells were significantly increased in the spleen at 7 days post-infection, and in the spleen and bone marrow at 25 days post-infection (Fig 7 and Table 3). NK cells are cytotoxic lymphocytes that kill virus-infected cells independently of MHC displayed viral antigens [103]. Additionally, NK cells secrete large amounts of cytokines (namely interferon (IFN)γ) that stimulate anti-viral immune responses [115]. The observed increase in NK cells could indicate that the immune system was mounting a potent anti-viral response. NK cell populations are elevated in humans infected with CHIKV, and these NK cells are associated with clearance of CHIKV-infected cells. However, elevation of NK cell populations persisting beyond 30 days post-infection is also associated with development of chronic arthritis in humans [10,116]. We observed elevated NK cell populations at 25 days post-infection and infected mice showed muscle/joint inflammation and reduced mobility. Because of this, the hu-NSG model may be appropriate for studying the effects of human NK cells on the development of arthritis following CHIKV infection.

Neutrophils were significantly increased in the blood and spleen at 7 days post-infection and significantly decreased in all tissues at 25 days post-infection (Fig 7 and Table 3). Neutrophils are phagocytic cells that eliminate pathogen/cellular complexes and kill extracellular pathogens through release of nitric oxide and genomic DNA nets. Furthermore, neutrophils are typically associated with inflammation and early immune response. CHIKV infections in non-humanized mice indicate that functional neutrophils contribute to clearance of CHIKV but also to the development of arthritis [117,118]. Neutrophil numbers increased during acute CHIKV infection in humans, but their specific role and the timing of the increase during the course of infection is unknown [119]. Since human neutrophil populations are modulated in hu-NSG mice infected with CHIKV, longer term studies could help clarify the role of these cells in producing arthritis-like signs of disease.

Taking all these data into account, it is clear that the hu-NSG mouse model was able to mount an immune response to CHIKV infection via mosquito bite. The changes in DCs, monocytes, Mϕ, immune cells (T-cells, B-cells, NK cells, neutrophils), and T-cell subsets (DP T-cells, NK T-cells) are consistent with immunological changes seen in humans, thus making the hu-NSG model relevant to further study CHIKV in humans.

## Discussion

This report presents a strong rationale for utilizing hu-NSG mice infected with a natural mosquito vector for studying human CHIKV pathogenesis and treatments. Though CHIKV injected mice demonstrated viremia, there was no significance in temperature, erythema, or histological findings as compared to uninfected animals. After infection via mosquito bite, mice demonstrated clinical signs similar to human disease (fever, erythema, and decreased activity), as well as viremia (RNA and infectious virus) in blood, immunological responses (human IgM and immune cell populations), and histologically relevant lesions (myositis, tendonitis, synovitis, bone marrow necrosis). In addition, the immune responses observed in these hu-NSG mice were due to their human immune system (as opposed to murine responses), as the markers used for both IgM and immune cell populations were human-specific.

While multiple mouse models have been used to study the pathogenesis and pathophysiology of CHIKV [26,32], none accurately model human disease. Most CHIKV models demonstrate either acute disease or chronic disease, but not both. Haese et al [25], Ganesan et al [28], and Teo et al [31] have reviewed multiple mouse models for studying acute versus chronic CHIKV disease. One acute disease model utilizes neonatal mice and mortality endpoints, with injection of large quantities of virus [120]. This is useful to determine the high susceptibility of neonates, but it is not helpful for extrapolating to disease in the general population. Another acute disease model uses mortality of immunocompromised mice, with genetically-modified mice with deficiencies in type I IFNs [121]. These mice are useful because they create a lethal model of disease, with systemic infection and mortality endpoints; however they are not helpful in determining pathogenesis mechanisms, because they lack innate immunity. Other studies have used young (14 day old) C57BL/6 mice injected into the footpad [30]; these animals have not yet matured and develop severe symptoms due to their immunocompromised status. Also, three day old BALB/c mice injected in the scruff developed viremia peaking at 10^6^ PFU on day 6, followed by death 7 days post-injection [122,123]. These studies detected specific genes that were upregulated, thus leading to increased severity and death. However, the conclusions are limited to the determination of the genes necessary for a young murine anti-viral response. Therefore, using the humanized mouse model described here, one could incorporate some of the genes detected in these preliminary studies, and determine if trends continue as the animals mature and the severity of disease changes.

Chronic disease models have utilized wild-type adult mice (C57BL/6 or BALB/c) [35,100,124–126] or immunocompromised adult mice (Rag1^-/-^) [34] that are injected in various routes (intra-articular, subcutaneous, intravenous). Though viral RNA persistence is seen in the joints after subcutaneous or intra-articular injection, isolation of infectious virus in these mice has been unsuccessful [30,34,35,124]. This makes it unclear as to whether clinical signs are due to the immune response to viral antigens or true viral replication and persistence. Other chronic models of disease focus on animal arthritis and/or myositis; in these models immunocompromised mice are injected with virus via the subcutaneous or intra-articular route, leading to limb/joint swelling at 3–7 days after infection [29,89]. This is useful for studying the chronic joint swelling seen with CHIKV chronic infections, but less helpful due to the absence of other clinical signs or systemic disease.

Hawman et al [127,128] described multiple CHIKV models used for studying pathogenicity, including the immunocompromised Rag1^-/-^ mouse (animals lacking an adaptive immune system) inoculated in the rear footpad, 3 week old C57BL/6 mice inoculated in the rear footpad, 6 week old wild type (C57BL/6) and CD4^-/-^ mice (animals deficient in CD4^+^ T-cells) inoculated in the subcutaneous region of the ankle, 3 day old BALB/C mice injected in the scruff, and other types of immunocompromised mice (including the NRG mouse, an animal deficient in B, T, and NK cells). Rag1^-/-^ mice had prolonged RNA viremia (up until 84 days post-inoculation) but no detection of infectious virus; they also had viral RNA in the liver up until 14 days, and inactive CHIKV (non-reproductive virus) remaining within the synovium, leading to synovitis [127,128]. Though helpful for the study of basic pathogenesis (emphasizing the importance of B and T cells), it can be difficult to determine if the clinical signs are from the disease itself or from residual viral antigens and/or the mouse’s inability to clear the pathogen due to its immunodeficient state. Three week old C57BL/6 mice inoculated in the footpad were shown to have viral RNA in the joints of the rear legs; however, the virions were determined to be non-infectious [124]. Again, this raises the question as to whether the virus itself, or simply the injection location, led to the clinical signs seen. Six week old C57BL/6 mice showed a viral peak and clearance within 10 days, while CD4^-/-^ mice demonstrated a higher peak and viremia up to 80 days post-injection [129]. Joint swelling (as measured by Vernier calipers) was more severe for 9 days (day 8–17 post-inoculation) in the immunodeficient mice [129]; however, since CD4^+^ T cells are not present in these animals, it is unclear if the prolonged joint swelling was due to localized inflammation that was slow to resolve or viral persistence. Another study comparing viremia and foot swelling among multiple strains of 6–12 week old immunodeficient mice (including the NRG mouse) was done after injecting 10^4^ CCID_50_ into each hind footpad. Viremia persisted (levels ranging 10^3^–10^5^ CCID_50_/mL) in mice that lacked T cells, indicating the necessity of T cells for the suppression of viremia [130], and foot swelling was reduced in NRG mice (as compared to C57BL/6 control mice). NRG mice did demonstrate signs of neurological disease as time progressed [130], a clinical sign that is sometimes seen acutely in humans. Again, although immunodeficient animals are good models that overcome mouse resistance to infection, clinical findings were not consistent with human disease (variable joint swelling, altered timing of disease symptoms).

When comparing any of the above models to the hu-NSG model described here, there is substantial evidence that the addition of the human immune system yields a more relevant model that recapitulates the timing and disease in human CHIKV infections. Up to now, mouse models have not represented the diverse CHIKV clinical outcomes seen in humans (acute vs. chronic; fatal vs. clinical or subclinical; young vs. old); however, the model described here showed a variety of outcomes (fatal, variable viremia with or without an IgM response, variable progression and severity of joint disease), thus allowing for the study of multiple aspects of the disease.

Our model demonstrates an accurate representation of cellular immunity as seen in humans upon CHIKV infection. We observed an overall increase in human monocytes/Mϕ (CD3^-^CD14^+^, especially the CD11c^-^ monocyte subpopulation) in the bone marrow (where these cells are generated) and decreases in the both CD11c^+^ and CD11c^-^ monocytes/Mϕ subpopulations in the blood at 7 days post-infection. Monocytes circulate in the bloodstream and can differentiate into Mϕ or DCs and migrate to various tissues to increase various cell populations during immune responses [103]. They serve as antigen-presenting cells, which phagocytose pathogens and present pathogen-associated peptides (in the context of MHC class II molecules) to B-cells and T-cells in the lymph node [103]. This results in activation of B- and T-cells, and generation of an adaptive immune response [103]. CD11c expression correlates with monocyte/Mϕ function; CD11c is typically upregulated during inflammatory conditions, recruiting these cells to the site of inflammation [131]. However, since we observed decreases in the monocyte/Mϕ population independent of CD11c expression, changes in the monocyte/Mϕ cell population are unlikely to be due to recruitment to sites of inflammation. The decrease in cell populations could be the result of cell death by direct CHIKV infection or migration of cells to other parts of the body. Future studies can determine the cause of this decrease in monocyte/Mϕ populations.

We also observed a decrease in total monocytes/Mϕ in the blood at 25 days post-mosquito bite with specific decreases in the following subpopulations: CD11c^+^ monocytes and CD11c^-^ monocyte/Mϕ in the blood; CD11c^+^ and CD11c^-^ monocytes in the spleen; and CD11c^+^ monocytes and CD11c^-^ monocytes/Mϕ in the bone marrow. Monocytes and Mϕ are susceptible to CHIKV infection [27,102] and may have gotten infected and succumbed to cell death in the hu-NSG mice over the course of our study. Based on our serum qRT-PCR data (Fig 3), CHIKV RNA persists in the blood of infected hu-NSG mice for at least 25 days. Since serum IFAs were positive, indicating infectious virus, it is plausible that CHIKV could still be infecting and decreasing monocyte/Mϕ populations at that time point. Another possibility is that these monocytes/Mϕ have migrated from the blood to another location, such as the joints and/or muscles. If these cells migrated to the joints and muscles, this could explain the myositis observed via histology (Fig 4) and IHC (Fig 5), as well as the reduced activity of the mice (Fig 2D). Synoviocytes, (specialized cells of the synovial joints) are susceptible to CHIKV infection, and they secrete large amounts of monocyte and Mϕ attractant chemokines, resulting in inflammation of the joints and arthralgia (e.g. IL-6, MCP-1) [132–134]. Additionally, monocytes and Mϕ have been shown to infiltrate the muscle in non-humanized mice, macaques, and humans infected with CHIKV, leading to myalgia [27,98,120]. Further studies will need to be done to determine whether the decrease in monocytes/Mϕ we observed was due to cell death or cell migration.

DCs are the primary antigen-presenting cell of the immune system and are key in initiating the adaptive immune response. These cells also direct the immune response to Th1 (anti-viral), Th2 (anti-parasitic), or Th17 (pro-inflammatory) responses through production and secretion of different cytokines. The increased number of myeloid DCs in CHIKV-infected hu-NSG mice may be an attempt to control the infection. However, DCs are susceptible to CHIKV infection themselves, and these increased cell numbers may effectively increase CHIKV replication [100]. Monocyte-derived DCs are tissue-resident cells whose populations expand during inflammatory conditions and produce cytokines to perpetuate inflammation [135]. These cell types are thought to potentiate joint inflammation in rheumatoid arthritis, and this inflammation is ameliorated, in part, through medications that inhibit differentiation of monocyte-derived DCs [136,137]. The role of these cells in CHIKV-mediated arthritis is unknown. Future studies could investigate the specific role these populations have in clearing the infection or perpetuating chronic arthritis/arthralgia.

The decrease in activated B-cells at 25 days post-infection is similar to observations in human CHIKV infections. B-cell populations in the blood are significantly decreased starting at day 1, and may continue decreasing until 60 days post-infection [102]. However, the specific functions of B-cells in the context of CHIKV infection are unclear. Studies in non-humanized mouse and rhesus macaque models indicate that functional B-cells and neutralizing antibodies are integral for combatting CHIKV infection, however these antibodies can also contribute to the development and persistence of arthritis [129,138–140]. Other studies in immunocompromised mice indicate that CHIKV becomes resistant to the B-cell response, allowing the virus to establish persistence [127]. Studies in hu-NSG mouse models could help elucidate whether these B-cells are protective or harmful during CHIKV infections.

There were no significant changes in CD4^+^ or CD8^+^ T cells at any time point in our hu-NSG mice study. Both CD4^+^ and CD8^+^ T-cells are involved in anti-viral immunity and during infection. Antigen-presenting cells (e.g. DCs, Mϕs) present viral antigens to CD4^+^ helper T-cells, thereby activating them [103]. Activated CD4^+^ T-cells stimulate CD8^+^ T-cells to mature into cytotoxic T lymphocytes, which kill virus-infected cells [141]. During this process, CD4^+^ and CD8^+^ T-cells that possess T-cell receptors specific to the viral antigen will proliferate [103]. Humans infected with CHIKV experience increased CD8^+^ T-cell populations starting as early as 1 day post-infection and lasting up to 10 weeks post-infection in CHIKV-induced chronic arthritis [142,143]. Therefore, we expected to observe an increase in these T-cells in hu-NSG mice infected with CHIKV. The hu-NSG mouse model does not produce robust human T-cell responses, because the mice lack a human thymus, resulting in human T-cells that recognize mouse antigen-presenting cells as opposed to human antigen-presenting cells [144]. Studies using humanized mouse models with robust human T-cell responses, such as the BLT model, could clarify the role of these cells in CHIKV pathogenesis.

Many varieties of humanized mice exist, each with a number of benefits and limitations [63]. We used NSG mice humanized through the Hu-SRC-SCID method due to the ease of producing engraftment with lower GVHD (as compared to the Hu-PBL-SCID method) [145]. Histological evaluation of tissues revealed a reasonable amount of subclinical GVHD, and flow cytometry revealed an incomplete immune system, in human T-cells, as mentioned above. These studies could be enhanced through the use of other humanized models, such as BLT or DRAG/NRG mice. These models use human thymic tissue (BLT model), allowing for a more robust engraftment and education of human immune cells, or use a different strain of mice that can form human antibodies capable of class-switching (DRAG model). Though technically and surgically challenging to produce, BLT model possess a more robust human immune system [64], and as such, would provide a unique milieu in which to test immunological parameters that may enhance CHIKV disease. No matter which model is used, any form of humanized mouse would allow for the study and characterization of neutralizing antibodies. With alphavirus infection of mice, viremia is typically no longer detectable at the point where neutralizing antibodies become detectable. In our studies, 50% of animals showed an IgM response while viremia was still present, indicating that the mice are profoundly immunosuppressed in comparison with normal animals. Neutralizing antibody titers were not determined here due to a lack of sufficient quantities of mouse serum, thus we could not compare IgG presence versus viremia. Since immunocompromised animals lack the ability to fully clear the infection, this model could be used to determine the relationship of human antibody responses alone to serum viremia.

Besides longer-term studies, more precise movement tests could be done on infected mice, to determine the dynamics of viral replication and onset of arthritis-like disease. Our results showed a decrease in wheel running activity in CHIKV-infected mice when compared to control mice. However, due to individual animal variability, statistically significant differences could not be determined. Further characterization of the decreased movement in animals through behavioral monitoring (such as telemetry-activated devices or video monitoring to detect wheel activity for specific animals) or coordination and muscle testing could help define this clinical correlate. Additionally, it could allow for further examination of time points within CHIKV disease when movement is impaired, and thus focus on the pathophysiology of the arthritis. IHC resulted in positive staining of affected muscle fibrils, so it is likely that the muscle degeneration was a direct result of CHIKV infection and cellular degeneration. Further characterization of this muscle loss could lead to a better understanding of the debilitating musculoskeletal illness seen during the chronic stages of disease in humans. Further diagnostics, such as measuring of joints using calipers, viral isolation with cell culture, and viral enumeration from muscles and/or joints (using qRT-PCR) could also be done to further define this clinical correlate.

Analysis of the cytokine/chemokine response present in these hu-NSG mice would further enhance the utility of this model. Cytokines are inflammatory mediators that balance and fluctuation during an immune response. High cytokine levels have been associated with inflammatory disease, including rheumatoid conditions and inflammatory joint disease in humans [146]. Retrospective analysis of human samples after an Italian CHIKV outbreak revealed an increase in IL-6 during the acute phase of infection, and low levels of IL-1β, TNF-α, IL-12, IL-10, IFN-γ, and IL-5 that increased as disease progressed to the chronic state [147]. Analysis after a Singaporean outbreak identified an increase in IL-1β and IL-6 with a decrease in RANTES could be used as an indicator of CHIKV disease severity [148]. It is unknown if these responses are due to the virus itself, the mosquito saliva, or both. Our research using the same hu-NSG mouse model demonstrated changes in human cytokine and chemokine levels in response to non-infected mosquito bites [43]. As such, further characterization of the immune response to infected mosquito bites could be done and compared to the latter, to separate out these effects.

The model reported here would be ideal for future preclinical studies, as it incorporates both human immune responses and the effects of mosquito delivery. Historically, preclinical vaccine studies using viral particles alone without including aspects of natural vector transmission, such as mosquito saliva, have been less likely to succeed in clinical studies [51]. By using an animal model that uses natural routes of transmission, with a realistic number of mosquitoes biting, we can increase the likelihood that disease pathogenesis is being observed authentically, thus increasing the likelihood of vaccine translation during the late phases of preclinical testing. Also, the human responses to varying strains and passages of CHIKV can be variable, leading to differences in attenuation and variation among studies [149]. Our model has the potential for studying the relevant viral strain variations that might occur in natural settings. Also, by incorporating human-based immune responses engrafted from human donors, the natural variability in human responses can be reproduced, thus potentially yielding more reliable results. One immediate application would be to test the efficacy of human anti-inflammatory therapies on long-term infected mice to reduce muscle and tendon pathology, in addition to bone marrow damage. These studies could lead to more appropriate treatments for patients developing severe CHIKV disease, thus reducing the most important public health burden of this virus.

## Supporting information

S1 Table Mice utilized for needle inoculation experiments. Data for humanized mice used in needle inoculation CHIKV-infection studies.
(TIFF)

S2 Table Mice utilized for mosquito bite inoculation experiments. Data for humanized mice used in mosquito bite CHIKV-infection studies.
(TIFF)

S3 Table Flow cytometry markers. Human immune system markers and reagents used for flow cytometry panels.
(TIFF)

S4 Table qRT-PCR. qRT PCR primer and probe sequences as adopted from Pastorino and colleagues (Pastorino B, Bessaud M, Grandadam M, Murri S, Tolou HJ, Peyrefitte CN. Development of a TaqMan RT-PCR assay without RNA extraction step for the detection and quantification of African Chikungunya viruses. J Virol Methods. 2005;124(1–2):65–71).
(TIFF)

S5 Table ELISA measuring IgM in the serum of mice infected with CHIKV by mosquito bite. ELISA values are based on a 450nm filter for humanized mice infected with CHIKV via mosquito bite. Samples were converted to standard units by multiplying the average sample absorbance by 10, and dividing by the cut-off value (run as part of the sample kit). Samples were considered positive if the absorbance value was greater than 10% over the cut-off value and negative if the absorbance value was less than 10% under the cut-off value; samples that were neither positive nor negative were deemed inconclusive/equivocal.
(TIFF)
