# Correction: Mosquito-bite infection of humanized mice with chikungunya virus produces systemic disease with long-term effects

**DOI:** 10.1371/journal.pntd.0010503

**Published:** 2022-06-02

**Authors:** Brianne M. Hibl, Natalie J. M. Dailey Garnes, Alexander R. Kneubehl, Megan B. Vogt, Jennifer L. Spencer Clinton, Rebecca R. Rico-Hesse

Fig 5 is incorrect. The authors have provided a corrected version here.

**Fig 5:**
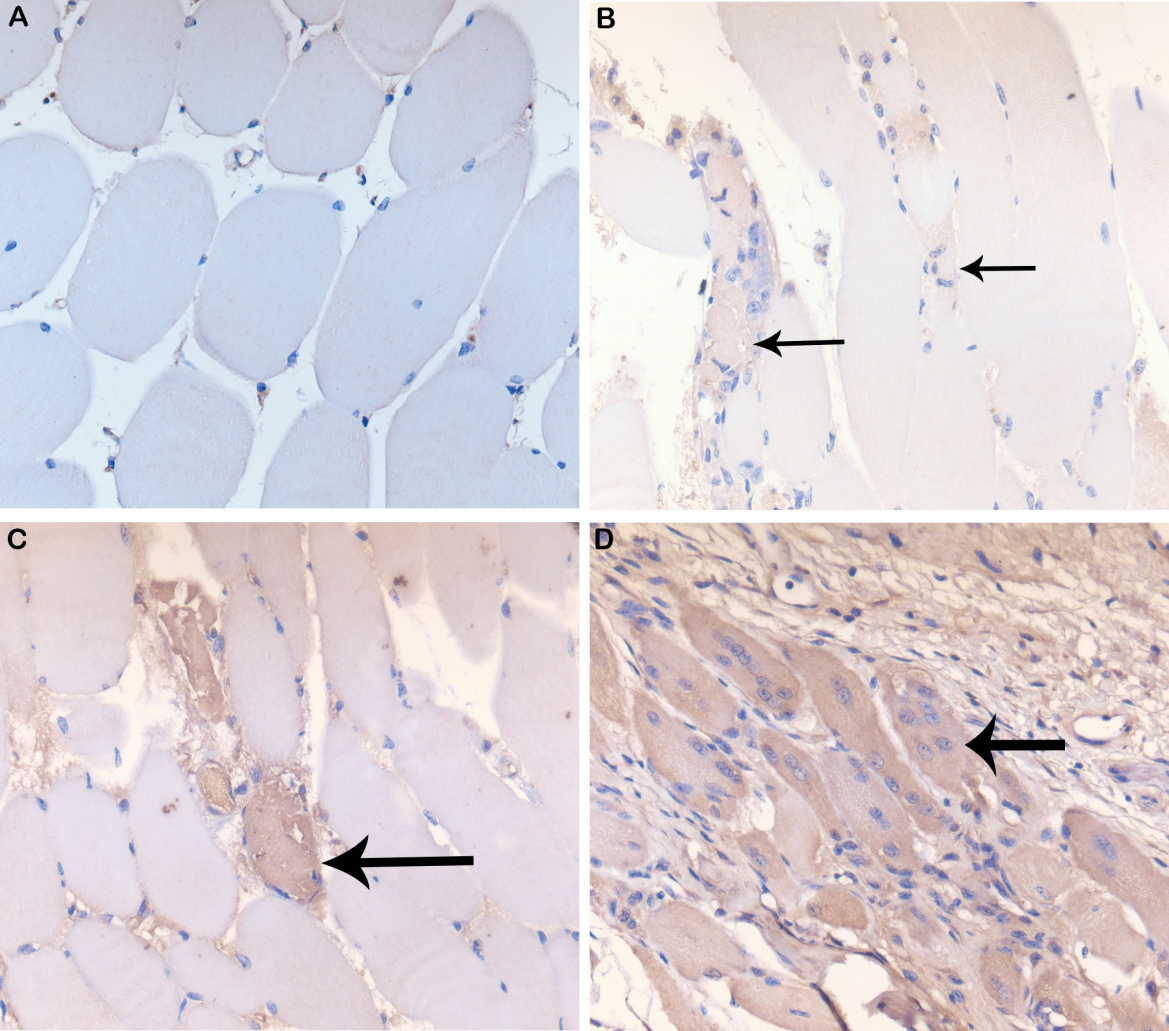
IHC of lesions in CHIKV-infected Hu-NSG mouse muscles. Immunohistochemistry (IHC) staining of CHIKV-infected mouse gastrocnemius muscle with CHK-263 antibodies. Positive staining (brown) represents CHIKV Env protein. **(A)** Control mouse with background IHC staining, 40X magnification. **(B)** Infected, degenerate muscle fibrils from minimal (score 1) myositis at day 7 post mosquito bite, 40X magnification. **(C)** Infected, degenerate muscle fibrils from mild (score 2) myositis at day 14 post mosquito bite, 40X magnification. **(D)** Infected muscle fibrils from moderate (score 3) myositis at day 21, 40X magnification.
