# Supplementary figures and images for: Mosquito-bite infection of humanized mice with chikungunya virus produces systemic disease with long-term effects
Source: PLoS Negl Trop Dis. 2021 Jun 9;15(6):e0009427. doi: 10.1371/journal.pntd.0009427 (PMC8189471; doi:10.1371/journal.pntd.0009427)

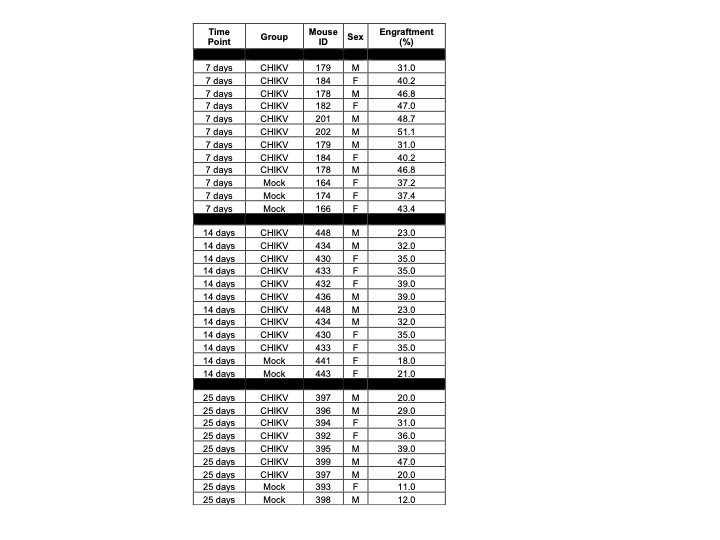

Supplement: S1 Table — Data for humanized mice used in needle inoculation CHIKV-infection studies. (TIFF) [file pntd.0009427.s001.tiff]

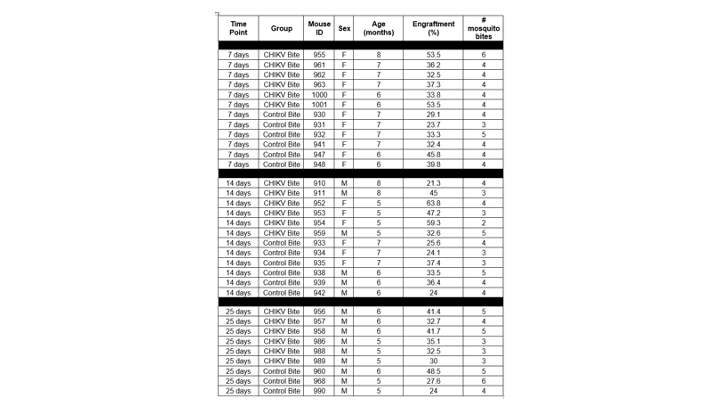

Supplement: S2 Table — Data for humanized mice used in mosquito bite CHIKV-infection studies. (TIFF) [file pntd.0009427.s002.tiff]

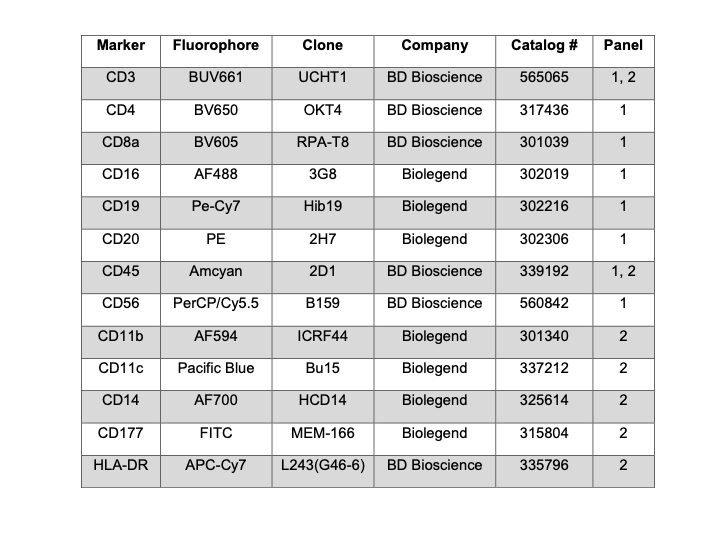

Supplement: S3 Table — Human immune system markers and reagents used for flow cytometry panels. (TIFF) [file pntd.0009427.s003.tiff]

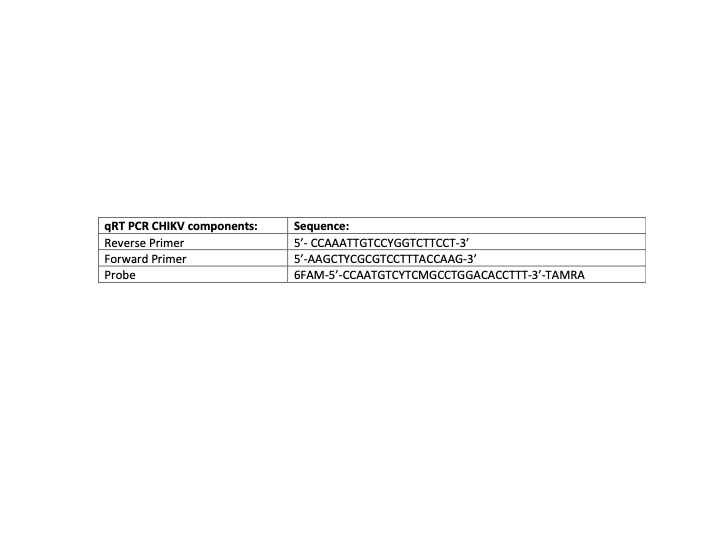

Supplement: S4 Table — qRT PCR primer and probe sequences as adopted from Pastorino and colleagues (Pastorino B, Bessaud M, Grandadam M, Murri S, Tolou HJ, Peyrefitte CN. Development of a TaqMan RT-PCR assay without RNA extraction step for the detection and quantification of African Chikungunya viruses. J Virol Methods. 2005;124(1–2):65–71). (TIFF) [file pntd.0009427.s004.tiff]

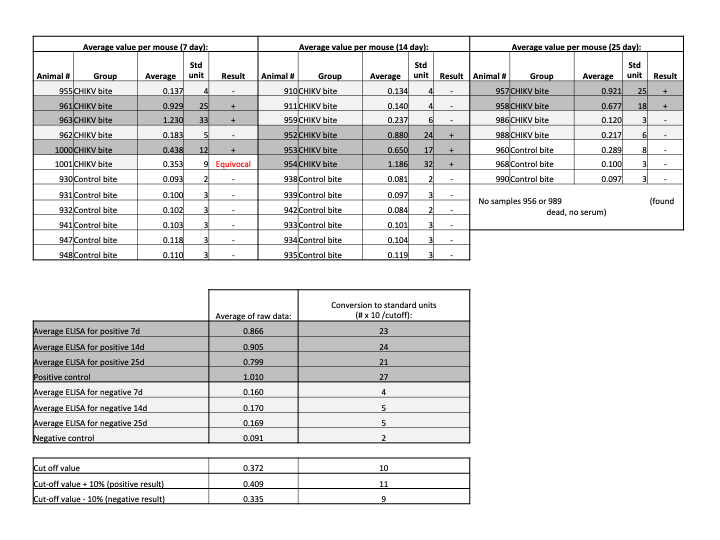

Supplement: S5 Table — ELISA values are based on a 450nm filter for humanized mice infected with CHIKV via mosquito bite. Samples were converted to standard units by multiplying the average sample absorbance by 10, and dividing by the cut-off value (run as part of the sample kit). Samples were considered positive if the absorbance value was greater than 10% over the cut-off value and negative if the absorbance value was less than 10% under the cut-off value; samples that were neither positive nor negative were deemed inconclusive/equivocal. (TIFF) [file pntd.0009427.s005.tiff]
